# Exploring a Pool‐seq‐only approach for gaining population genomic insights in nonmodel species

**DOI:** 10.1002/ece3.5646

**Published:** 2019-09-26

**Authors:** Sara Kurland, Christopher W. Wheat, Maria de la Paz Celorio Mancera, Verena E. Kutschera, Jason Hill, Anastasia Andersson, Carl‐Johan Rubin, Leif Andersson, Nils Ryman, Linda Laikre

**Affiliations:** ^1^ Division of Population Genetics Department of Zoology Stockholm University Stockholm Sweden; ^2^ Science for Life Laboratory and Department for Biochemistry and Biophysics Stockholm University Solna Sweden; ^3^ Department of Medical Biochemistry and Microbiology Uppsala University Uppsala Sweden; ^4^ Department of Animal Breeding and Genetics Swedish University of Agricultural Sciences Uppsala Sweden; ^5^ Department of Veterinary Integrative Biosciences Texas A&M University College Station TX USA

**Keywords:** genetic diversity, genome sequencing, population genomics, *Salmo trutta*, salmonid, single nucleotide polymorphism

## Abstract

Developing genomic insights is challenging in nonmodel species for which resources are often scarce and prohibitively costly. Here, we explore the potential of a recently established approach using Pool‐seq data to generate a de novo genome assembly for mining exons, upon which Pool‐seq data are used to estimate population divergence and diversity. We do this for two pairs of sympatric populations of brown trout (*Salmo trutta*): one naturally sympatric set of populations and another pair of populations introduced to a common environment. We validate our approach by comparing the results to those from markers previously used to describe the populations (allozymes and individual‐based single nucleotide polymorphisms [SNPs]) and from mapping the Pool‐seq data to a reference genome of the closely related Atlantic salmon (*Salmo salar*). We find that genomic differentiation (*F*_ST_) between the two introduced populations exceeds that of the naturally sympatric populations (*F*_ST_ = 0.13 and 0.03 between the introduced and the naturally sympatric populations, respectively), in concordance with estimates from the previously used SNPs. The same level of population divergence is found for the two genome assemblies, but estimates of average nucleotide diversity differ ($\bar{\pi }$ ≈ 0.002 and $\bar{\pi }$ ≈ 0.001 when mapping to *S. trutta* and *S. salar*, respectively), although the relationships between population values are largely consistent. This discrepancy might be attributed to biases when mapping to a haploid condensed assembly made of highly fragmented read data compared to using a high‐quality reference assembly from a divergent species. We conclude that the Pool‐seq‐only approach can be suitable for detecting and quantifying genome‐wide population differentiation, and for comparing genomic diversity in populations of nonmodel species where reference genomes are lacking.

## Graphical abstract

We explore the potential of a newly established approach that relies on Pool‐seq data to generate de novo genome assemblies in nonmodel species upon which Pool‐seq data are used to estimate population divergence and diversity. Estimates of population differentiation between sympatric brown trout (*Salmo trutta*) populations are qualitatively similar to those found from a previously used SNP panel and highly concordant to estimates from mapping our data to the publicly available Atlantic salmon (*Salmo salar*) reference genome. The Pool‐seq only approach is deemed suitable for detecting population differentiation in nonmodel species for which reference genomes are lacking.

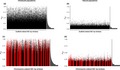

## 1 INTRODUCTION

Understanding the importance of genetic variation for species' persistence continues to be a major research goal in population genetic and evolutionary studies (Allendorf & Ryman, 2002; Bernatchez, 2016; Soulé & Wilcox, 1980). Quantifying genetic variation within and among populations is important for conservation and management (Fuentes‐Pardo & Ruzzante, 2017; Ovenden, Berry, Welch, Buckworth, & Dichmont, 2015; Volckaert, 2015), and for understanding mechanisms of local adaptation, hybridization, and introgression (Allendorf & Hard, 2009; Waples, Punt, & Cope, 2008).

Developing population genetic insights from enough markers to represent whole genomes quickly and cost‐effectively is challenging in nonmodel species (Schlötterer, Tobler, Kofler, & Nolte, 2014). An alternative to whole‐genome sequencing (WGS) for such species is subsampling of the genome, which provides insights into genome‐level variation at comparably lower cost per individual, thereby enabling assessment across more individuals (Davey et al., 2011; Gagnaire, Pavey, Normandeau, & Bernatchez, 2013; Martinez, Buonaccorsi, Hyde, & Aguilar, 2017; Wang, Shashikant, Jensen, Altman, & Girirajan, 2017). Another alternative, while staying at the genome‐wide scale, is the pooling of individuals for WGS. This enables the sampling of many chromosomes per base pair and thereby accurate estimates of the site frequency spectrum, at a low individual cost (Kofler, Langmüller, Nouhaud, Otte, & Schlötterer, 2016).

Reference genomes provide crucial information needed for organizing, orienting, and annotating WGS reads. When there is none available, as is the case for many nonmodel organisms, the assembly of a closely related species is often used. Relatedness between focal and reference species has implications for the representation of markers found (Recknagel, Jacobs, Herzyk, & Elmer, 2015). As an alternative to using reference genomes of closely related species or for situations when no such reference is available, we here apply a newly developed exon mining via Pool‐seq approach to acquire a draft genome assembly both for the focal species and for single nucleotide polymorphism (SNP) frequency estimation. The approach leverages the power of Pool‐seq to subsample the genome to obtain high‐resolution genomic insights quickly and at a reasonable cost (Neethiraj, Hornett, Hill, & Wheat, 2017). This pipeline has been utilized for elucidating the genomics underlying phenotypic differences between populations of several butterfly species (Keehnen, Hill, Nylin, & Wheat, 2018; Pruisscher, Nylin, Gotthard, & Wheat, 2018; Woronik & Wheat, 2017).

Pool‐seq data generate highly fragmented assemblies, and in order to reduce fragmentation, the method explored here uses transcriptome data from the same species to scaffold contigs that are annotated to nonoverlapping regions of the same protein. The genome assembly is subsequently reduced to only contain scaffolds with identified and unique gene models (including introns and untranslated regions). Pool‐seq data from populations are mapped against the final gene‐models‐only genome assembly which contains both protein‐coding and noncoding sequences, for estimation of population diversity and differentiation (akin to mapping RNA‐seq data against a de novo transcriptome of the same data). This method (Neethiraj et al., 2017) has similarities with that of Therkildsen and Palumbi (2017) who mapped Pool‐seq reads to a reference transcriptome. In our case, we generate a transcriptome from published RNA‐seq data and use it to scaffold and annotate a draft genome assembly from Pool‐seq data.

We explore the Pool‐seq‐only approach of Neethiraj et al. (2017) using the brown trout (*Salmo trutta*) which belongs to the family Salmonidae that is characterized by large genomes (c. 3 Gbp) with the added complexity of a whole‐genome duplication event that occurred roughly 90 million years ago (MYA) followed by subsequent, and ongoing, rediploidization (Berthelot et al., 2014; Lien et al., 2016; Nugent, Easton, Norman, Ferguson, & Danzmann, 2017). Currently, there are genome assemblies available for Atlantic salmon (*Salmo salar*; Davidsson et al., 2010; Lien et al., 2016), rainbow trout (*Oncorhynchus mykiss*; Berthelot et al., 2014), chinook salmon (*Oncorhynchus tshawytscha*; Christensen, Leong, et al., 2018; Narum, Genova, Micheletti, & Maass, 2018), Arctic charr (*Salvelinus alpinus;* Christensen, Rondeau, et al., 2018), coho salmon (*Oncorhynchus kisutch*; GenBank assembly accession: GCA_002021735.1), and grayling (*Thymallus thymallus*; Sävilammi et al., 2019). The separation of brown trout and its closest relative the Atlantic salmon occurred c. 6–7 MYA (Pustovrh, Snoj, & Bajec, 2014), and nucleotide divergence between the two is below 2% (Leitwein et al., 2017). However, chromosomal rearrangements (Leitwein et al., 2017), number of chromosomes (*S. trutta* 2*n* = 80, Phillips & Ráb, 2001; *S. salar* 2*n* = 54–58, Brenna‐Hansen et al., 2012), and degrees of residual tetrasomy (Lien et al., 2016) differ significantly between the two species.

Similar to other species of Salmonidae, the brown trout is highly substructured (Laikre, 1999; Lerceteau‐Köhler, Schliewen, Kopun, & Weiss, 2013; Ryman, 1981; Ryman, Allendorf, & Ståhl, 1979; Vøllestad, 2018). Genetically distinct populations maintain separation across limited geographic areas (Palmé, Laikre, & Ryman, 2013; Stelkens, Pompini, & Wedekind, 2012), and the disparity of habitats occupied by brown trout has enabled population differentiation along a variety of phenotypic axes (Hansen, 2002; Hindar, Ryman, & Utter, 1991; Meier, Hansen, Bekkevold, Skaala, & Mensberg, 2011; Meier et al., 2014; Palm & Ryman, 1999). Understanding the role of genetic variation for sustainable management and conservation monitoring is crucial for this socioeconomically important species (Charlier, Laikre, & Ryman, 2012; Hansen, Ruzzante, Nielsen, & Mensberg, 2000; Leitwein, Gagnaire, Desmares, Berrebi, & Guinand, 2018; Petereit et al., 2018).

Our aim is to explore the potential of an exon mining through Pool‐seq approach to characterize the genomic variation and differentiation among brown trout populations. We ask whether this approach is suitable for answering population genomics questions by studying two pairs of sympatric populations for which we can make well informed hypotheses based on previous work (Andersson, Jansson, et al., 2017; Andersson, Johansson, Sundbom, Ryman, & Laikre, 2017; Palm & Ryman, 1999; Palmé et al., 2013). One pair of populations is naturally and cryptically sympatric while the other consists of two experimentally released populations, and we expect differentiation to be greater among the latter. We use Pool‐seq data from one of these populations to generate a de novo brown trout assembly, and then map the Pool‐seq data to this reference to estimate pool‐specific diversity and pairwise differentiation. These results are compared to the differentiation found from previous analyses of the same populations using allozymes and SNPs (Andersson, Jansson, et al., 2017; Andersson, Johansson, et al., 2017; Palm & Ryman, 1999; Palmé et al., 2013). We further contrast the outcome from mapping the Pool‐seq data to our draft assembly to mapping against the reference genome of a related species by repeating the analyses for Pool‐seq data mapped to an available Atlantic salmon genome (Lien et al., 2016).

## 2 MATERIALS AND METHODS

### 2.1 Populations studied

**Figure 1:**
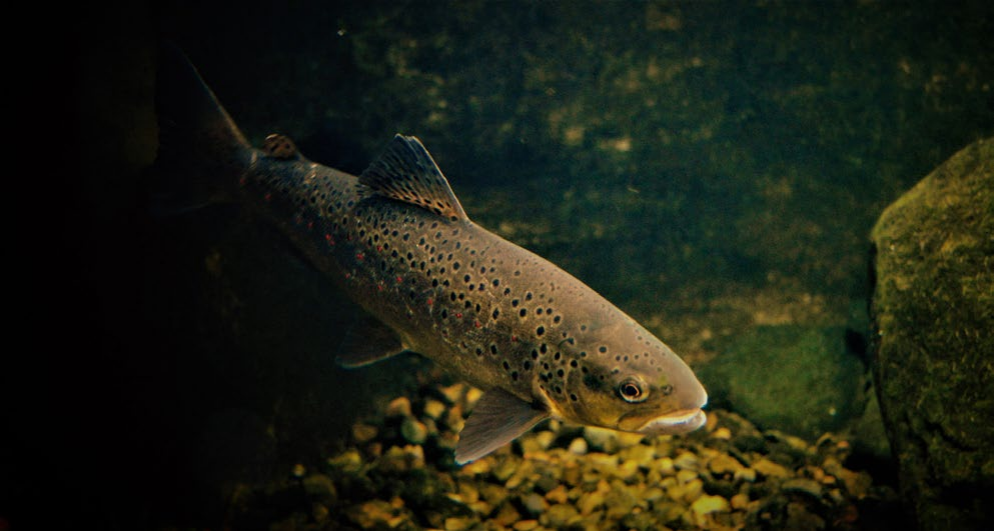
The brown trout (*Salmo trutta*) from Swedish mountain lakes was used as a case study to explore the potential of a recently presented Pool‐seq‐only approach for gaining genomic insights in nonmodel species. Photograph by Anastasia Andersson

**Figure 2:**
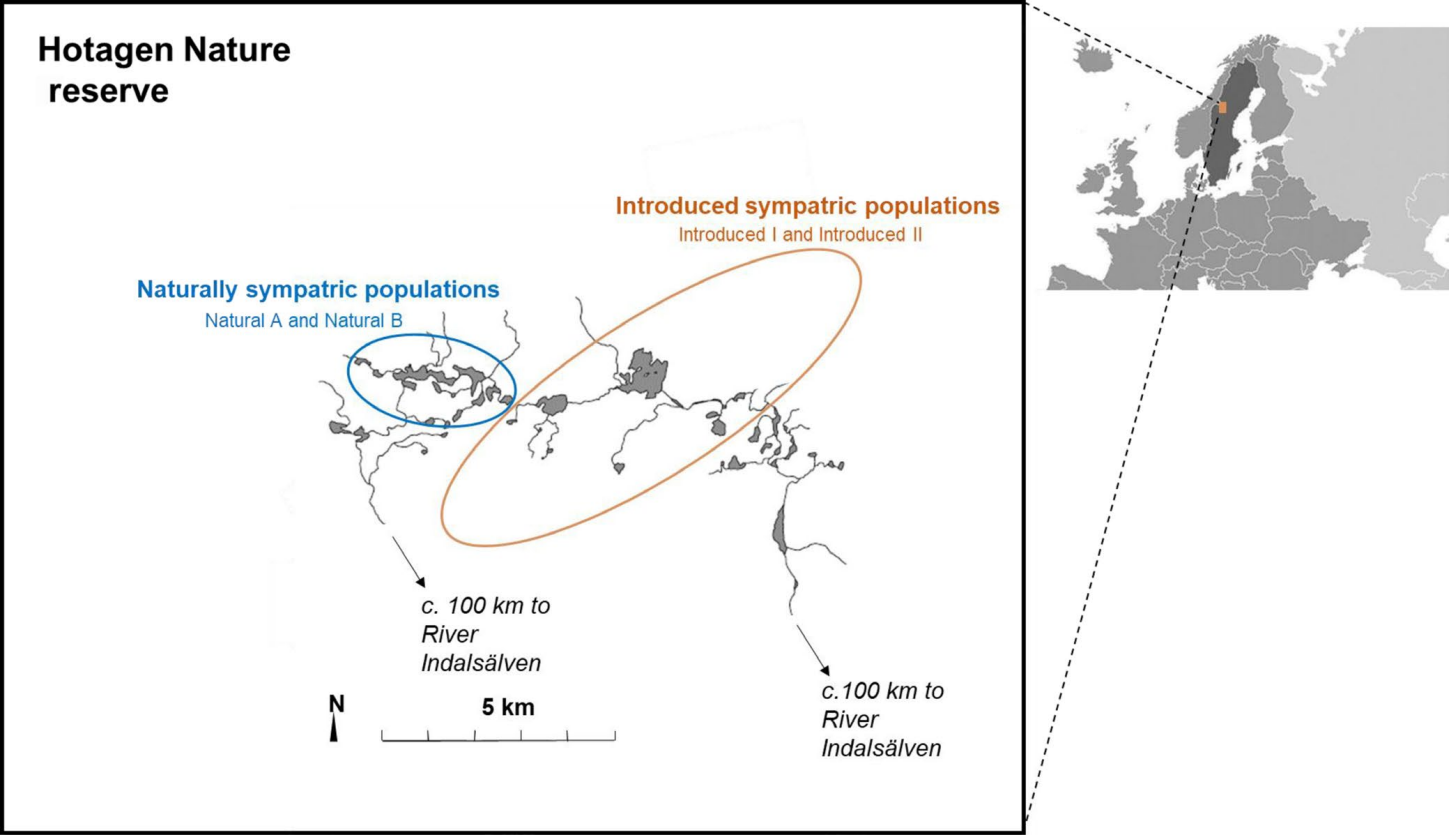
Map of study sites located in Hotagen Nature Reserve, Sweden. Circles indicate sampled lakes inhabited by introduced and naturally sympatric populations, respectively. Both waters are connected to the River Indalsälven which drains into the Baltic Sea c. 400 km from the study site

Two pairs of sympatric brown trout (Figure 1) populations inhabiting small freshwater lakes in the mountainous regions of the County of Jämtland, central Sweden, were studied. One pair consists of two populations that co‐occur in a natural setting due to an artificial release to this environment whereas the other pair is comprised of naturally sympatric populations. The first pair of populations was collected from the lake system Bävervattnen (Figure 2, Appendix S1, Figure S1) into which fish had been introduced as fry in 1979 for experimental purposes. The released individuals were from two separate populations that had been isolated from each other since the last glaciation (c. 5,000–9,000 years ago) and were ecologically diverged and genetically marked by contrasting homozygosity at the allozyme locus *AGP‐2* (Palm & Ryman, 1999). This pair will be referred to as the *introduced populations I* and *II*. The second pair of populations comes from the lake system Trollsvattnen (Figure 2, Appendix S1) where the two main lakes are inhabited by a population pair that has previously been described as cryptically sympatric because no phenotypical or ecological difference between them has been possible to detect in spite of extensive screenings (Andersson, Jansson, et al., 2017; Andersson, Johansson, et al., 2017; Palmé et al., 2013). Their existence was initially detected through a consistent heterozygote deficiency at multiple allozyme loci (Jorde & Ryman, 1996), later validated by extensive allozyme monitoring showing consistent population divergence as measured by the fixation index (*F*_ST_) over several decades (Palmé et al., 2013) and by a 3 K SNP panel (Andersson, Jansson, et al., 2017). This pair of populations will henceforth be referred to as the *natural populations A* and *B*.

### 2.2 Samples

We used *n* = 50 individuals from each of the two introduced populations (Palm & Ryman, 1999) caught during 1988–1995; frozen tissue had been stored since collection. The fish were assigned to either of the introduced populations based on their allozyme genotype at the marker locus *AGP‐2* and their age based on otolith readings. Only individuals representing the parental generation (*P*) or the *F1* generation for which population assignment (no hybrids) was possible using the *AGP‐2* genotype were considered. A total of 41 individuals representing the *P* generation were available, and 59 *F1* fish were randomly selected out of c. 700 available fish to provide *n* = 50 per population.

The natural populations have been monitored for many years with access to thousands of fish. We used *n* = 50 individuals per natural population collected during 2002–2007 and assigned to their respective population by Andersson, Jansson, et al. (2017) based on a STRUCTURE (Falush, Stephens, & Pritchard, 2003; Pritchard, Stephens, & Donnelly, 2000) analysis from 14 polymorphic allozyme loci and an assignment score above 0.8 (for more details, see Appendix S1).

### 2.3 DNA extraction, library preparation, and sequencing

DNA was isolated from muscle tissue that had been stored at −80°C since sampling 1–3 decades ago using Qiagen's DNeasy Blood and Tissue Kit according to the manufacturer's protocol (Qiagen) with an additional RNase A treatment. DNA quality was assessed by visual inspection of DNA fragmentation on agarose gels and absorbance at 260/280. DNA with high molecular weight from each of 50 individuals per population was quantified using fluorometry (Qubit; Thermo Scientific) and pooled at equal concentrations to achieve 3 μg pooled genomic DNA in a volume in the range of 65–120 μl. The National Genomics Infrastructure (NGI), Uppsala, Sweden (Science for Life Laboratory), conducted the construction of PCR‐free paired‐end libraries with an average insert size of 350 bp (TruSeq) followed by sequencing using read length 150 bp on an Illumina HiSeq 2000 machine.

### 2.4 Short‐read data preparation and de novo assembly

Illumina short reads from the four population pools were cleaned for adapters and low‐quality bases using BBDuk implemented in BBTools version 37.31 (http://sourceforge.net/projects/bbmap/). Pool‐seq short‐read data from natural population A were used to generate a draft de novo genome assembly using CLC Genomics version 5.5.1 with default settings: k‐mer size 20, bubble size 50, and minimum contig length 200.

### 2.5 Transcriptome assembly

We used publicly available RNA‐seq data (Carruthers et al., 2018; https://www.ncbi.nlm.nih.gov/sra/SRX3421649%5Baccn%5D) from whole organism tissue of hatchery strain juveniles of undetermined sex (Table S1) to generate a *S. trutta* transcriptome. The paired‐end reads, from 8 accessions available at the time of study prior to the official release of the *S. trutta* transcriptome, were cleaned for adapters in BBMap's implementation version 37.31 (http://sourceforge.net/projects/bbmap/) following default recommendations. Rcorrector version 2 (Song & Florea, 2015) was used at default settings to further filter the data for singleton kmers and ribosomal RNA filtered by BBDuk in the BBTools suite version 37.53 (http://sourceforge.net/projects/bbmap/). The resulting data were used as input for a transcriptome assembly using Trinity version 2.5.1 (Grabherr et al., 2011) with default parameters. The transcriptome assembly was then collapsed into unique protein sequences using the Evigene software (Gilbert, 2013). In order to assess its quality, we compared this unique protein set from *S. trutta* to the Atlantic salmon protein sequence predictions from the available genome assembly (Lien et al., 2016; accession number GCF_000233375.1). Before analysis, salmon protein sequences were collapsed using CD‐hit into clusters of 90% identity, keeping only the longest member of each cluster for subsequent analysis; this is hereafter referred to as the salar90 protein dataset. This approach is identical to the clustering of UniRef (Suzek, Huang, McGarvey, Mazumder, & Wu, 2007) database to make the UniRef90 dataset, and in this case, it allows us to keep only the longest isoforms and only single members of recent gene duplications. By comparing the *S. trutta* protein sequences against this salar90 protein dataset, we quantitatively assessed how many genes we assembled compared to expected numbers. We determined whether each gene was assembled at partial or full length by dividing the length of the assembled *S. trutta* protein by the length of the salmon homolog identified using BLASTP (*e*‐value cutoff 10e‐5; protein–protein BLAST; version 2.2.28+).

### 2.6 Scaffolding, annotation, and quality assessment of the Pool‐seq de novo genome assembly

Scaffolding involves joining contigs that belong to nonoverlapping regions of the same protein and thus reduces assembly fragmentation. The Pool‐seq de novo assembly was scaffolded using the *S. trutta* protein sequences via the MESPA pipeline (Neethiraj et al., 2017). MESPA uses the software SPALN version 2.1.4 (http://www.genome.ist.i.kyoto-u.ac.jp/~aln_user/spaln/; Gotoh, 2008) for protein to genome alignment, and then uses this output to guide further scaffolding per protein based on exons from a single protein that are located on different scaffolds. Gene models for the resulting superscaffolded assembly are thus based on the *S. trutta* protein dataset. To avoid complications in mapping and variant calling caused by the partially tetraploid characteristics of the *S. trutta* genome, we used a collapsed version of the full Pool‐seq de novo assembly containing only scaffolds with identified gene models, and of those only retained regions with unique gene models. We used this haploid assembly for subsequent analyses of SNPs within or near our gene model annotations. The completeness of this draft *S. trutta* assembly was assessed based on gene content from near‐universal single‐copy orthologs using BUSCO version 1.22 (Simão, Waterhouse, Ioannidis, Kriventseva, & Zdobnov, 2015), with the library of ray finned fish proteins (*Actinopterygii*; *Danio rerio*) and default settings.

### 2.7 Read mapping and quality filtering

Paired‐end short reads from each pool filtered for adapters and minimum base quality 20 were mapped to the draft *S. trutta* assembly and the Atlantic salmon genome (Lien et al., 2016; accession number GCF_000233375.1). We tested three different mapping algorithms: BBMap version 37.31 (http://sourceforge.net/projects/bbmap), bwa mem available in bwa version 0.7.17 (Li & Durbin, 2009), and NextGenMap (NGM) version 0.5.4 (Sedlazeck, Rescheneder, & Von Haeseler, 2013). The mapping success for each algorithm was assessed in Qualimap version 2.2.1 (García‐Alcalde et al., 2012) before and after filtering the bam files at varying levels of mapping quality. Based on these comparisons (Table S2) and visual inspection of bam files in the Integrative Genomics Viewer (IGV; Robinson et al., 2011), bwa mem and filtering for mapping quality 20 were chosen for all subsequent analyses, trading off evenly distributed read coverage across the assembly with mapping accuracy.

Bam files with short‐read data mapped to the two genome assemblies were filtered to keep only properly paired reads. Mpileup files were generated from these bam files using samtools version 1.6 (Li et al., 2009), filtering for mapping quality 20 and base quality 20, as well as invoking the parameter “‐B” to reduce false SNPs from misalignments. The mpileup files obtained from mapping to the *S. trutta* assembly were inspected for insertions or deletions (indels) using the identify‐genomic‐indel‐regions.pl script in POPOOLATION2 version 1201 (Kofler, Pandey, & Schlötterer, 2011). Indels and 5 bp downstream and upstream every indel were subsequently removed using the filter‐pileup‐by‐gtf.pl tool from POPOOLATION2. Coverage with reads mapped to the *S. salar* reference genome was highly uneven. Therefore, indels ≥32,766 bp had to be removed from the mpileup file using a custom script before running the scripts implemented in POPOOLATION2 which cannot handle indels of this size and larger. Read depth histograms were assessed for each bam file to define minimum and maximum depth thresholds for subsequent population genetic calculations (Appendix S2).

### 2.8 Population genomic analyses

Population genomic variation was assessed for each pool using POPOOLATION version 1.2.2 (Kofler, Orozco‐ter Wengel, et al., 2011), including estimates of nucleotide diversity (*π*; Tajima, 1983) which quantifies the degree of polymorphisms at a locus within a population and Tajima's D (*T*_D_), which measures deviations from mutation–drift equilibrium at segregating sites due to selection or demographic events (Tajima, 1989). Since estimates of *π* and *T*_D _are sensitive to sequencing errors (Kofler, Orozco‐ter Wengel, et al., 2011), we subsampled each mpileup file to uniform coverage based on depth histograms (targeting the mode of each pools' coverage distribution and omitting sites with coverage exceeding the mode +½ of the mode; Appendix S2) by running the subsample‐pileup.pl script without replacement (Kofler, Orozco‐ter Wengel, et al., 2011). The script Variance‐sliding.pl implemented in POPOOLATION was used to detect SNPs from subsampled mpileup files and to simultaneously calculate *π* (including invariant sites) and *T*_D_. Calculations were made for nonoverlapping 500‐bp windows across each of the assemblies, using a minor allele count of 2 for a SNP to be called and stringent depth filters for variant and invariant sites to be included (the mode of each pools' depth distribution ±½ of the mode; Appendix S2, Figures S2 and S3). Only windows of >90% coverage with data after applying depth and minor allele frequency filters were included in the analyses. All summary statistics were calculated and statistical tests were performed in R (R Core Team, 2017). A Kruskal–Wallis rank‐sum test for independence of *π* and *T*_D_ with respect to populations was performed. If the null‐hypothesis of samples coming from the same distribution was rejected, Wilcoxon rank‐sum tests were performed between all pairs of populations with *p*‐values adjusted using Bonferroni correction. The scripts used for the population genomic analyses are available in Appendix S3.

The fixation index (*F*_ST_; Nei, 1973) was estimated for each population pair in POPOOLATION2 version 1201 (Kofler, Pandey, et al., 2011). First, indel‐filtered mpileup files (not subsampled) were converted to the POPOOLATION2‐specific sync format using the script mpileup2sync.jar. *F*_ST_ was calculated using the script fst‐sliding.pl with the following parameters, while simultaneously detecting SNPs. Variant and invariant sites with a read depth lower or higher than the thresholds identified from read depth histograms (the mode of the coverage distribution ±½ of the mode; Appendix S2) were excluded from the analyses, and a minor allele count of 3 was used as cutoff for a SNP to be called. *F*_ST_ was estimated for nonoverlapping windows to avoid increased stochastic error rates associated with small window size (Kofler, Pandey, et al., 2011). A range of window sizes (1 bp–5 kb) and window coverages with data after applying all quality filters (0.5–1.0) were tested before choosing a window size of 500 bp and restricting the analysis to windows of >90% coverage with data after applying depth and minor allele frequency filters (Appendix S2, Table S3). Using the same parameters as described above, *F*_ST_ was also obtained for noncoding and coding regions, the latter of which to enable a fairer comparison to results obtained from previously published allozyme data. A Kruskal–Wallis rank‐sum test for independence of pairwise *F*_ST_ values from the full assembly, coding regions, and noncoding regions was performed.

### 2.9 Comparison to *F*_ST_ estimates from previously used markers

We compared *F*_ST_ values from Pool‐seq data mapped to the *S. trutta* assembly to previous divergence estimates using SNP genotyping of individuals from the same populations (*n* = 2,832 SNPs genotyped for 18 individuals and *n* = 2,852 SNPs genotyped for 30 individuals from each of the introduced and natural populations respectively; Andersson, Jansson, et al., 2017; L. Laikre & N. Ryman, unpublished data). All *n* = 18 individuals from each of the introduced populations were also included in the Pool‐seq samples from those populations. Similarly, the majority of the *n* = 30 individuals genotyped previously for the natural populations were included in the pools for those populations (overlap of *n* = 27 and *n* = 28 for natural populations A and B, respectively). We identified the putative locations of these SNPs in the *S. trutta* assembly by blasting the sequence surrounding each SNP to the *S. trutta* assembly and retaining the best hits (BLASTN; version 2.2.28+). For each location, we retrieved the corresponding *F*_ST_ value estimated in POPOOLATION2 per base pair for Pool‐seq data using BEDTools intersect version 2.25.0 (Quinlan & Hall, 2010). *F*_ST_ per locus for already available SNP data from individual genotyping had previously been calculated in GENEPOP version 4.0.7 (Rousset, 2008) but was recalculated here using Nei's (1973) *F*_ST_ = 1−*H*_S_/*H*_T_ since this *F*_ST_ was used by POPOOLATION2 for the Pool‐seq data according to the manual. We also compared global *F*_ST_ estimates from allozymes and SNPs to our assembly‐wide and window‐based averages.

## 3 RESULTS

### 3.1 Transcriptome assembly

RNA‐seq data from whole organism tissue of hatchery strain juveniles were used (Carruthers et al., 2018) and removal of singleton kmers and rRNA from these raw data reduced the total number of paired‐end reads by c. 6%, resulting in a total of 188 million (M) paired‐end reads (on average 47 M paired‐end reads per accession). The initial transcriptome assembly (254,432 contigs with an N50 of 1,779 bp and an average contig length of 963 bp) was reduced to 82,052 protein‐coding contigs with an N50 of 1,077 bp, average contig length of 600 bp, and total length of 49 Mbp in the final *S. trutta* protein dataset. Seventy‐eight percent of complete Actinopterygian core genes were recovered in our *S. trutta* transcriptome assembly. A total of 12,711 (35%) of the proteins in the salar90 protein dataset were represented by nearly full‐length (>90%) sequences in our *S. trutta* protein dataset.

### 3.2 De novo assembly of Pool‐seq data

Protein‐based superscaffolding of the *S. trutta* assembly reduced fragmentation, as reflected in a reduced number of contigs and increased N50 (Table 1). Before scaffolding, we recovered 63,636 *S. trutta* protein‐coding gene predictions including isoforms at near full length (>90%), and this number increased to 70,376 postscaffolding. A total of 87,191 unique, that is, single‐copy, gene models were identified in the final, collapsed *S. trutta* assembly. These gene models were located on in total 38,888 scaffolds with an N50 of 17,722 bp, and a total length of 446,412,000 bp (Table 1).

**Table 1:** Genome assembly statistics for the de novo assembly of natural pool A from CLC Genomics version 5.5.1 and for the superscaffolded assemblies from MESPA (Neethiraj et al., 2017)

Metric	Prescaffolding genome assembly	Postscaffolding MESPA genome assemblies	
	CLC assembly	Full assembly	Gene‐models‐only assembly
*n* (contigs)	1,096,446	1,085,382	38,888
N50 (bp)	5,944	6,194	17,722
Total contigs length (bp)	1,847,698,765	1,848,805,165	446,412,000
Percentage (%) of non‐ATGC characters^a^	0.77	0.83	0.57

Note

The number (*n*) of contigs is specified but refers to the number of scaffolds in the annotated MESPA genome assemblies where contigs have been joined to form scaffolds. The gene‐models‐only assembly is the *S. trutta* genome assembly used for population genomics in the present study.

a Non‐ATGC characters: for example, ambiguous nucleotides or unknown nucleotides (*N*).

Of the 4,584 Actinopterygian single‐copy orthologs, the initial *S. trutta* assembly had 58% complete (45% single‐copy and 14% duplicated), 20% fragmented, and 22% missing orthologs. After superscaffolding, 71% of matches were complete (59% single, 12% duplicated), 12% fragmented, and 17% missing.

### 3.3 Processing of short‐read data for population genomic analyses

On average, 69 giga base pairs (Gbp) of raw data were generated per population pool, corresponding to c. 9 M reads. After cleaning, 30% of reads (on average 2.8 M read pairs) mapped to the *S. trutta* gene models as proper pairs and c. 50% of reads mapped as pairs against the *S. salar* reference (on average 5.0 M read pairs; Table 2). Coverage of depth was lower for reads mapped to the *S. salar* reference (mode of depth distribution: ~50× to *S. trutta*, and ~45× to *S. salar*), which is nearly 6 times the size of the *S. trutta* assembly, and its annotation contains 97,918 gene model predictions. Although reads had been filtered for mapping quality 20, average mapping quality was 11 for reads mapped to *S. salar* due to the large proportion of reads that did not map (Table 2; mapping quality histograms in Figure S4). The edit distance between the reads and the reference was similar for both reference genomes (2.6%–2.7% and 2.9%, respectively; Table 2).

**Table 2:** BAM file statistics from Qualimap version 2.2.1 (García‐Alcalde et al., 2012) for Pool‐seq data from each of the four *S. trutta* population pools filtered for minimum base quality 20 and mapped to the generated *S. trutta* assembly and the previously available *S. salar* assembly, respectively, using bwa mem and mapping quality 20

Population pool	Introduced I	Introduced II	Natural A	Natural B
	*S. trutta*			
Reference size (bp)	446,412,000	446,412,000	446,412,000	446,412,000
Number of reads mapped as pairs	259,292,315	254,225,047	280,138,464	325,530,950
Percentage of reads mapped as pairs	29.8%	29.7%	30.3%	30.5%
Mode of depth of coverage	47	46	50	57
Mean mapping quality	55	56	56	55
General error rate^a^	2.6%	2.6%	2.6%	2.7%

	*S. salar*			
Reference size (bp)	2,966,890,203	2,966,890,203	2,966,890,203	2,966,890,203
Number of reads mapped as pairs	468,965,008	457,736,395	497,860,918	573,473,218
Percentage of reads mapped as pairs	54.3%	53.8%	54.2%	54.0%
Mode of depth of coverage	40	41	45	50
Mean mapping quality	11	11	11	11
General error rate^a^	2.9%	2.9%	2.9%	2.9%

Note

The mode of depth of coverage was obtained from BEDTools version 2.25.0 (Quinlan & Hall, 2010).

a General error rate is estimated from the ratio of total collected edit distance to the number of mapped bases.

### 3.4 Population genomic analyses

On average, 53,538 SNPs were called in POPOOLATION from quality‐filtered and subsampled mpileup files for each pool mapped against our constructed *S. trutta* assembly (Table 3), and 231,548 SNPs on average when mapping to the *S. salar* reference genome, taking depth (Appendix S2) and minor allele frequency filters into account for each site. Average nucleotide diversity, $\bar{\pi }$, ranged between 1.65 and 1.95 with the *S. trutta* reference (Table 3; Figure S5). Although several confidence intervals overlap, the distributions differ significantly among populations (Kruskal–Wallis *H *= 145.26, *df *= 3, *p *< 2.2e‐16) with the lowest value observed in introduced population II. Pairwise comparisons from Wilcoxon signed‐rank tests showed that all population pairs except that of introduced population I versus natural population A differed, to a degree that results in statistical significance. With the *S. salar* reference genome, average $\bar{\pi }$ were considerably lower—between 0.97 and 1.14 (Table 3)—but here too the lowest variability is noted in introduced population II and the population distributions differ (*H *= 2,916.7, *df*= 3, *p *< 2.2e‐16); all pairwise comparisons provided statistically significant differences among all population pairs.

**Table 3:** Average nucleotide diversity ($\bar{\pi }$) and Tajima's D (*T*_D_) with 95% confidence intervals in brackets estimated from Pool‐seq data mapped to the *S. trutta* and *S. salar* assemblies, respectively

Reference	Pool	*n (*windows)	*n *(SNPs)	$\bar{\pi }$ (10^–3^)	*T*_D_
*S. trutta*	Introduced I	16,346	52,473	1.86 (1.81–1.92)	−0.065 (−0.081 to −0.048)
	Introduced II	15,564	45,783	1.65 (1.60–1.70)	−0.180 (−0.200 to −0.163)
	Natural A	17,250	55,707	1.80 (1.74–1.84)	−0.160 (−0.174 to −0.141)
	Natural B	16,212	60,188	1.95 (1.90–2.01)	−0.202 (−0.220 to −0.184)
*S. salar*	Introduced I	125,934	204,956	1.07 (1.06–1.08)	0.103 (0.098–0.108)
	Introduced II	127,524	200,006	0.97 (0.96–0.98)	−0.038 (−0.043 to −0.033)
	Natural A	155,326	289,247	1.14 (1.13–1.15)	−0.004 (−0.009 to 0.001)
	Natural B	120,600	231,984	1.09 (1.08–1.10)	−0.030 (−0.036 to −0.024)

Note

Average values were obtained from estimates of nonoverlapping, 500‐bp windows with >90% coverage after subsampling and quality and depth filtering. *n *(windows): total number of windows; *n *(SNPs): total number of SNPs.

Tajima's D values (*T*_D_) were all below 0 ranging from −0.202 to −0.065 when mapping to *S. trutta* with the largest value observed in introduced population I (Table 3, Figure S6); several confidence intervals overlap, but we do find differences between distributions (*H* = 228.3, *df* = 3, *p *< 2.2e‐16). Pairwise comparisons indicate statistically significant differences between the populations except for between introduced population II and each of the natural populations. When mapping to *S. salar*, *T*_D_ increases substantially, but the relationship with the largest value observed for introduced I remains (Table 3). Here too, the distributions of *T*_D_ among the three populations with lower *T*_D_ overlap, but with significant differences among all four distributions (*H *= 2,818.6, *df *= 3, *p *< 2.2e16) and statistically significant differences in *T*_D_ distributions between both population pairs.

**Figure 3:**
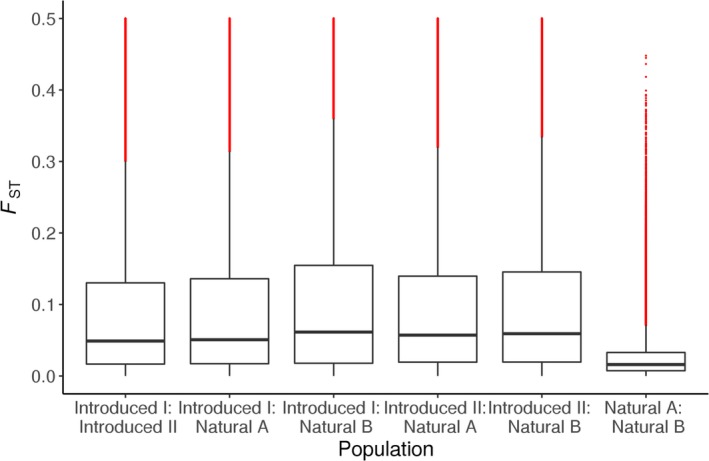
Boxplot of *F*_ST_ values within 500‐bp windows along the *S. trutta* assembly for all pairwise comparisons between population pools. The horizontal line at the center of the box is median *F*_ST_, and the top and bottom of the box show 25th and 75th percentiles, respectively. Vertical black lines show the boundaries of the interquartile range and red markings show outliers

**Figure 4:**
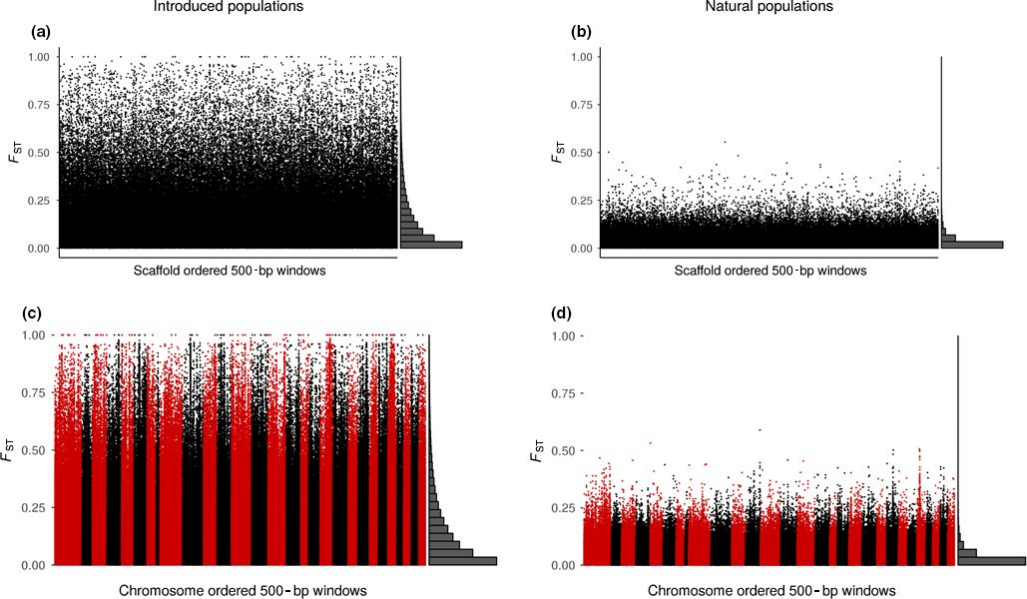
Pairwise *F*_ST_ values within 500‐bp windows along the (a, b) *S. trutta* and (c, d) *S. salar* assemblies for (a, c) introduced and (b, d) naturally sympatric populations. Histograms in the margins represent frequency distributions of *F*_ST_ values

We used POPOOLATION2 for *F*_ST_ estimation, and an average of c. 1,500,000 SNPs were called assembly‐wide and per population comparison from quality‐filtered sync files for pools mapped to the *S. trutta* assembly (Table 4), taking depth (Appendix S2) and minor allele frequency filters into account per site. We compared all possible population pairs (six in total) and found average *F*_ST_ ≈ 0.10 for 5 of these comparisons, while one population pair—the two natural populations—showed *F*_ST_ = 0.03 (Figure 3, Table 4). The distribution of *F*_ST_ for the two main pairs of interest—the introduced versus the naturally sympatric populations—illustrates the considerably higher divergence for the introduced as compared to the natural pair (Figure 4a,b). Average *F*_ST_ was higher across coding (c. 11,000 SNPs) than noncoding (c. 1,000,000 SNPs) regions (introduced pair: *W* = 260 × 10^6^; *p* ≪ .001; natural pair: *W *= 263 × 10^6^; *p *≪ .001), although the mean values differed only slightly for each pair (Table 4).

**Table 4:** Average pairwise *F*_ST_ between introduced and natural populations, respectively

Marker	*n*	Pairwise *F*_ST_ between introduced populations	Pairwise *F*_ST_ between natural populations
Allozymes	14	0.41^a^	0.30^b^
SNP genotyping	*n* (introduced) = 2,832	0.34^a^	0.03^b^
	*n* (natural) = 2,852		
Pool‐seq mapped against *S. trutta*			
Assembly‐wide	*n* (introduced) = 1,466,801	0.1280 (0.1274–0.1285)	0.0276 (0.0275–0.0278)
	*n* (natural) = 1,525,838		
Coding regions	*n* (introduced) = 10,667	0.1491 (0.1436–0.1547)	0.0317 (0.0304–0.0330)
	*n* (natural) = 10,637		
Noncoding regions	*n* (introduced) = 1,083,064	0.1270 (0.1264–0.1277)	0.0274 (0.0273–0.0276)
	*n* (natural) = 1,126,527		
Pool‐seq mapped against *S. salar*			
Assembly‐wide	*n* (introduced) = 2,206,076	0.1548 (0.1544–0.1552)	0.0320 (0.0319–0.0321)
	*n* (natural) = 2,510,827		
Coding regions	*n* (introduced) = 24,936	0.1499 (0.1466–0.1533)	0.0326 (0.0318–0.0334)
	*n* (natural) = 25,676		
Noncoding regions	*n* (introduced) = 1,967,836	0.1544 (0.1540–0.1548)	0.0318 (0.0317–0.0319)
	*n* (natural) = 2,250,772		

Note

Estimates from previous studies using allozymes and SNP genotyping are given, as well as estimates from Pool‐seq data mapped to each of the *S. trutta* assembly and *S. salar* genome using nonoverlapping, 500‐bp windows with >90% coverage after subsampling, quality and depth filtering. The number (*n*) of allozyme markers, previously used SNPs, and Pool‐seq SNPs used in each pairwise comparison is specified. 95% confidence intervals for Pool‐seq estimates are given in brackets.

a L. Laikre and N. Ryman (unpublished data) using Weir and Cockerham's (1984) *F*_ST_.

b *F*_ST_ estimates between natural populations originally published in Andersson, Jansson, et al. (2017) and Weir and Cockerham's (1984) *F*_ST_.

*F*_ST_ estimates when mapping the Pool‐seq data to the *S. salar* reference were similar to those observed using the *S. trutta* assembly (using on average 2,400,000 SNPS; Table 4), and the distribution of *F*_ST_ remained higher for the introduced than the natural pair (Figure 3c,d). For both references, *F*_ST_ for the naturally sympatric population pair was close to 0.03, and for the introduced pair, *F*_ST_ was around 0.13 and 0.15 when mapping to the *S. trutta* and *S. salar* assembly, respectively. With the sort of “pseudochromosomal” context that *F*_ST_ windows were placed in from the transitory structures formed when using the *S. salar* reference, outlier regions appear more evident than when using the *S. trutta* assembly (cf. Figure 4a,b,c,d).

### 3.5 Comparison to *F*_ST_ estimates from previously used markers

**Figure 5:**
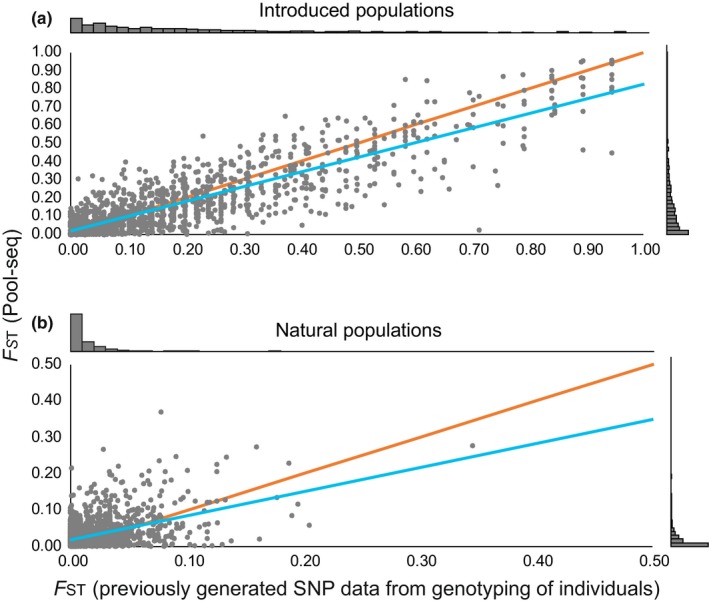
*F*_ST_ values from Pool‐seq analyses (*y*‐axes) compared to those from previous individual SNP genotyping of the same SNP loci (*x*‐axes; data from Andersson, Jansson, et al., 2017 and L. Laikre & N. Ryman, unpublished data). Nei's *F*_ST_ computed as *F*_ST_ = 1−*H*_S_/*H*_T_ was used for the previous SNP data, while for Pool‐seq, Nei's *F*_ST_ was computed using POPOOLATION v. 1.2.2. (a) Pairwise *F*_ST_ for 1,415 SNP loci for the introduced population (linear regression coefficient *b* = 0.81, *r ^2^*= 0.76, *t *= 68, *p *< 0.001). (b) Pairwise *F*_ST_ for 1,378 SNP loci for the naturally sympatric populations (linear regression coefficient *b* = 0.66, *r* ^2^ = 0.21, *t* = 19, *p* < 0.001). The blue lines are linear regression trend lines, and the orange ones represent expected values with *r^2 ^*= 1. Histograms in the margins represent frequency distributions of *F*_ST_ values

Estimates of *F*_ST_ from the previously used allozymes exceeded Pool‐seq averages calculated across assemblies, coding regions, and noncoding regions for both population pairs (Table 4). Average *F*_ST_ calculated across the previously used SNPs exceeded Pool‐seq *F*_ST_ for the introduced pair, while for the natural pair, the two estimates were concordant (Table 4). For our comparison of previously used SNPs with Pool‐seq SNPs, 1,974 and 1,985 of the 2,832 and 2,852 SNPs previously genotyped in the introduced and natural population pair, respectively (Andersson, Jansson, et al., 2017), were successfully located in our *S. trutta* assembly. Of these, *F*_ST_ estimates were available from the Pool‐seq data for 1,415 and 1,378 individual SNPs per population pair for the introduced and natural populations, respectively. *F*_ST_ values obtained from previous individual SNP genotyping and from Pool‐seq data were correlated for both population pairs (introduced: linear regression coefficient *b *= 0.81, *r* ^2^ = 0.76; *t *= 68; *p *< .001; natural: linear regression coefficient *b *= 0.66, *r* ^2 ^= 0.21; *t *= 19; *p* < .001; Figure 5).

## 4 DISCUSSION

### 4.1 Pool‐seq draft assembly and *S. trutta* transcriptome assembly

The *S. trutta* genome was assembled by superscaffolding a highly fragmented de novo assembly of Pool‐seq short‐read data, and simultaneously annotated using publicly available transcriptome data from the same species, using a newly established method for nonmodel organisms with limited genomic resources (Neethiraj et al., 2017). This assembly was used as a reference to map the Pool‐seq data and estimate population genomic metrics, both within and nearby protein‐coding regions. We used publicly available RNA‐seq data recently published in Carruthers et al. (2018) to assemble a draft *S. trutta* transcriptome. The validation of completeness of our *S. trutta* transcriptome, wherein 78% of single‐copy Actinopterygian orthologs were found, is comparable to recovery ranges of Carruthers et al. (2018), implying that the *S. trutta* transcriptome is suitable to annotate and identify coding regions in our final *S. trutta* assembly. We found nearly 13,000 *S. salar* proteins represented by nearly full‐length transcripts having >90% coverage in the final *S. trutta* transcriptome. This corresponds to c. 35% of all *S. salar* proteins. In their brown trout transcriptome, Carruthers et al. (2018) achieved c. 40% recovery of transcripts at near to full length from the same NCBI protein database for Atlantic salmon. This affirms good coverage and quality of our transcriptome assembly.

To improve mapping and subsequent variant calling, we used a haploid subset of the full *S. trutta* assembly, comprised only of contigs with gene model predictions. This reduced fragmentation compared to the full assembly is reflected in increased N50 (Table 1) and the representation of over 70 percent complete Actinopterygian core genes which is furthermore comparable to reports for the *S. salar* reference (Carruthers et al., 2018).

The study species used to explore the present Pool‐seq approach is a salmonid and thus has a genome duplication background. Although the duplication event is old—c. 90 million years—full rediploidization has not occurred (Lien et al., 2016), but we consider the MESPA approach to be careful in this respect since the method implies that only one copy of each contig with a gene model prediction of the original genome assembly remains in the final assembly and related contigs are grouped together (Neethiraj et al., 2017). Also, we were careful to apply stringent quality filters to the short‐read data mapped to the *S. trutta* draft assembly to avoid potential problems. Such problems could, for example, arise in duplicated regions that are only present as one copy in the *S. trutta* draft assembly. In such a region, short reads from both duplicates would map, including reads from the paralog region, which can be reduced with stringent mapping quality and read depth filters.

### 4.2 Population genomics analyses for pools mapped to the *S. trutta* assembly

We found no strong indications of demographic perturbations or selection in the sampled populations, which agrees with our general knowledge of these populations. Average nucleotide diversity estimated from data mapped to the *S. trutta* assembly was about 0.16%–0.20% across the four populations (Table 3, Figure S5). This estimate is lower than previous reports for brown trout ($\bar{\pi }$ ≈ 0.5%; Leitwein et al., 2016). Estimates of Tajima's D fell slightly below zero for all populations. Positive *T*_D_ indicates a greater degree of heterozygosity given the number of segregating sites, as expected after population contraction, while negative values of *T*_D_ may indicate population expansion after a recent bottleneck, though slightly negative values of *T*_D_ are expected for natural populations (Gillespie, 2004). We did find differences in both nucleotide diversity and *T*_D_ among populations, but differences are relatively small. Most pronounced is the lower level of nucleotide diversity of introduced population II which originates from a small mountain lake (0.14 km^2^) at the uppermost part of a water system, and where introduced fish originated from few parent fish caught in the wild and taken to a hatchery for production of fish for release.

Average *F*_ST_ between the two natural populations was the lowest of all pairwise comparisons, while the highest *F*_ST_ was found between the two introduced populations (Table 4, Figure 4). Genomic comparisons of sympatric populations where gene flow may occur are often distinguished by low diversity with condensed regions of divergent outliers (Jacobs et al., 2018), which may be indicated for the naturally sympatric pair (Figure 4), although we did not perform an outlier analysis in the present study. The comparably high divergence between the introduced populations was expected based on the source populations' geographic separation. Introduced population I stems from a hatchery population characterized by large piscivorous individuals with potential for long‐distance migration, whereas the source of the introduced population II is characterized by small, lake‐resident fish. One generation after introduction, descendants of introduced I migrated further downstream than descendants of introduced II (Palm & Ryman, 1999), indicating maintenance of the source populations' local adaptations. This, along with slightly higher values of *T*_D_, which was positive for introduced I when mapping pools to the *S. salar* reference, could again be indicative of the disparate genetic background and history of the introduced populations. A deeper understanding of the demographic and selective forces that have shaped the genomic variation of these populations requires further investigation.

### 4.3 Comparison to *F*_ST_ from SNP genotyping

One of the main benefits of Pool‐seq is the generation of vast amounts of SNPs per genome for entire populations, with the prospect of improving power of population detection and delineation (Anand et al., 2016). However, average differentiation based on the previously identified SNPs was inflated compared to average estimates based on all Pool‐seq SNPs in the introduced population pair, but not in the natural population pair (Table 4). Such a discrepancy has been found in other studies, for example, in pooled versus individually genotyped SNPs using RAD‐seq (Gaughran et al., 2018) and has multiple possible explanations. Importantly, the previously used SNPs have been selected based on high degree of polymorphisms, while our Pool‐seq includes loci with low minor allele frequencies. Secondly, individually genotyped SNPs are associated with ascertainment biases (Albrechtsen, Nielsen, & Nielsen, 2010; Lachance & Tishkoff, 2013) and the previously used SNPs were developed based on polymorphisms in brown trout of Danish breeding programs and Norwegian rivers (Andersson, Jansson, et al., 2017).

**Figure 6:**
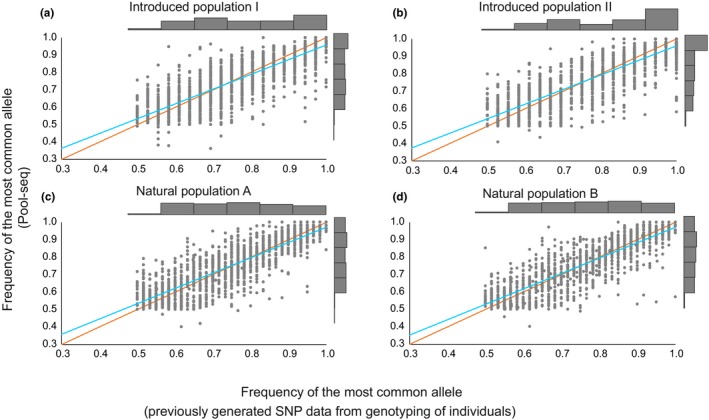
Comparison of frequency of most common alleles of individual SNP loci estimated using Pool‐seq data (*y*‐axes) versus previously genotyped individuals (*x*‐axes). (a) Frequency of the most common allele at each of 1,415 individual SNP loci for the introduced population *I* (linear regression coefficient *b* = 0.85, *r* ^2^ = 0.71, *t *= 60, *p *< 0.001), and (b) introduced population *II* (linear regression coefficient *b* = 0.84, *r* ^2^ *^^*= 0.76, *t *= 66, *p *< 0.001). (c) Frequency of the most common allele for each of the 1,378 individual SNP loci for the naturally sympatric populations A (linear regression coefficient *b* = 0.88, *r* ^2^ *^^*= 0.73, *t *= 60, *p *< 0.001) and (d) B (linear coefficient *b* = 0.88, *r* ^2^ *^^*= 0.74, *t *= 63, *p *< 0.001). Blue lines are linear regression lines, and orange lines represent expected values. The number of individuals was *n *= 50 for each of the pools and *n *= 18 for previous data from individual genotyping of the SNP loci for each of the introduced populations, and *n *= 30 for each of the naturally sympatric populations. Historgrams in the margins represent distributions of allele frequencies

In contrast to assembly‐wide average comparisons of *F*_ST_, the correlation of *F*_ST_ values from previously genotyped SNPs in individuals and the same SNPs in our present Pool‐seq data identified using BLAST showed high consistency between the two methods for the introduced pair, but a weaker correlation for the natural pair where variance in *F*_ST_ is low. For both pairs, we observe on average somewhat higher *F*_ST_ values with the individual genotyped SNPs (*x*‐axes; Figure 5) than from Pool‐seq *F*_ST_ values (*y*‐axes; Figure 5). Previous work has used allele frequencies directly to compare Pool‐seq approaches to conventional individual genotyping (Dorant et al., 2019; Hivert, Leblois, Petit, Gautier, & Vitalis, 2018; Zhu, Bergland, González, & Petrov, 2012), and when we compare frequency of the most common allele in each of the SNP loci from the previous individually based genotyping versus those from the pools, we find very strong correlation in all four populations (Figure 6a–d); coefficients of determination (*r* ^2^) are over 0.7 in all four populations and linear regression coefficients are well over 0.8 and close to 0.9 for each of the naturally sympatric populations. We suggest that observed differences (Figure 6) are largely due to the small sample sizes of the previous individually genotyped dataset of only *n *= 18 individuals for each of the introduced populations and *n *= 30 for each of the natural, sympatric pair. Although the same individuals are predominantly included in the pools, the additional individuals in the pools are likely to affect allele frequency estimates. The consistent observation of on average higher frequencies of the most common allele in the individually based dataset when allele frequencies approach 1, but lower allele frequencies than for the pools when allele frequencies are close to 0.5 is consistent with expectations from smaller sample sizes. Small sample sizes are expected to result in larger allele frequency estimates when the frequency of the most common allele is close to 1, and a contrasting discrepancy at lower allele frequencies. Our observations are in line with this expectation with the smaller sample sizes from the previously genotyped SNPs showing smaller values (Figure 6, *x*‐axes) of frequency of the most common allele than the allele frequency estimates from Pool‐seq (Figure 6, *y*‐axes) when close to 0.5 but the opposite when the frequency of the most common allele is close to 1 (compare blue regression lines to orange expected lines in Figure 6). Dorant et al. (2019) observe somewhat better correlations of allele frequencies from Pool‐seq versus conventional genotyping by sequencing than we do. However, they have larger sample sizes (*n *= 48) in both pools and individual sequencing and use the same individuals in both.

Average assembly‐wide *F*_ST_ from Pool‐seq data for both pairs of populations were much smaller than the estimates obtained from allozymes (Table 4). Our present data indicate that the previously used allozymes and SNPs do not reflect genome‐wide estimates of average divergence for the introduced populations. Further, our data support expectations and previous findings using SNP markers for the present naturally sympatric populations, namely that relatively few loci appear to be involved in this cryptic substructuring (Andersson, Jansson, et al., 2017). The genomic characteristics of partly reproductively isolated populations are expected to be primarily determined by drift and migration (Jacobs et al., 2018), probably reflected by the previously used SNPs and the assembly‐wide average *F*_ST_ from Pool‐seq data for the natural pair, and by diversifying selection in limited regions as represented by the allozymes (Andersson, Jansson, et al., 2017). The contention that only a few regions are under selection is further supported by the assembly‐wide patterns of divergence estimated from Pool‐seq data for the natural populations, which showed few sparsely placed peaks of limited differentiation (Figure 4b,d). The loci and genes involved in differentiation of both population pairs remain to be explored, and the present study has provided a tool with which a multitude of SNPs can be detected across the genome.

### 4.4 Comparison of *S. trutta* and *S. salar* references

To validate results obtained based on the *S. trutta* assembly, we mapped the Pool‐seq data to the Atlantic salmon reference genome (Davidsson et al., 2010; Lien et al., 2016). The relatedness between a resequenced species and the species for which a reference genome is available for mapping has implications for the representation of the resequenced genome (Recknagel et al., 2015). Nucleotide divergence between the Atlantic salmon and brown trout is below 2% (Leitwein et al., 2017). However, since the two *Salmo* species differ in chromosomal number and structure, as well as degree of tetrasomy, limited mapping success may be expected, for example, due to structural differences (Brenna‐Hansen et al., 2012; Lien et al., 2016). Thus, greater divergence between reads and reference is expected when mapping to the *S. salar* genome than to the *S. trutta* assembly, especially since the *S. trutta* assembly was made from one of the populations analyzed here – the naturally sympatric population A. Indeed, we find differences in mapping success when comparing the two reference genomes (Table 2). In spite of the larger sequence divergence, a higher percentage of reads mapped to the *S. salar* genome than to the *S. trutta* assembly. This may be explained by the level of completeness of the two genome assemblies. While the *S. salar* genome is a highly contiguous assembly at chromosome level (Lien et al., 2016), the *S. trutta* assembly is reduced to scaffolds harboring gene models that occurred in only one copy, representing c. 20% of the expected size of the *S. trutta* genome. Reads from genome regions not present in the *S. trutta* assembly may not map at all or map to the wrong region in the assembly, the latter of which will inflate coverage when mapping to the *S. trutta* reference.

There are also implications of relatedness between resequencing data and reference assembly for estimates of population genomic variation. Average nucleotide diversity (*π*) estimates were lower when using the *S. salar* reference than when mapping pools to the *S. trutta* assembly (Table 3). This might be attributed to the fact that we are expected to map reads to highly conserved regions between the two species (*S. trutta* vs. *S. salar*) and the conserved regions are likely to be less variable. The regions with most sequence diversity, on the other hand, are likely to be those in the process of divergence between the species. Reads mapped to the *S. trutta* draft assembly are expected to align with higher probability to those regions and may thus be better at reflecting nucleotide diversity. Similarly, the *T*_D_ values were larger when mapping to the *S. salar* reference compared to our *S. trutta* assembly. However, the relative estimates of both *π* and *T*_D _among pools are largely consistent for both references (Table 3).

Divergence estimates were highly concordant when comparing pools mapped to the two references (introduced populations assembly‐wide *F*_ST_ = 0.13 and 0.15 for *S. trutta* and *S. salar* references, respectively, and natural populations assembly‐wide *F*_ST _= 0.03 in both cases; Table 4). We were also able to place the *F*_ST_ results into a “pseudochromosomal” context when using the *S. salar* genome, which revealed clustered outlier SNPs in the Manhattan plots that were not as apparent when using the *S. trutta* assembly (Figure 4). We have not pursued outlier analyses here, but plan to return to this issue in forthcoming work. Finally, it is important to note that the genomes we studied here are large and complex and further population genomic studies using the Pool‐seq‐only approach applied here are warranted.

## 5 CONCLUSIONS

We explored a recently presented Pool‐seq‐only approach to generate a draft genome assembly for a nonmodel species. We then mapped Pool‐seq data to this assembly to estimate population genomic parameters. We used the brown trout (*S. trutta*) and two pairs of populations from which we had previous population genetic information from allozymes and individually genotyped SNPs. We also mapped our pools to a high‐quality reference genome from Atlantic salmon (*S. salar*), a closely related species, to compare to our Pool‐seq‐only results. We find high consistency in genome‐wide *F*_ST_ values between the two population pairs using the Pool‐seq‐only approach versus the *S. salar* reference. We find less consistency when comparing the genome‐wide Pool‐seq *F*_ST_ values to those obtained from 14 allozymes and those from c. 3,000 SNPs. In contrast, a high correlation in *F*_ST_ values and allele frequencies is observed when comparing the exact same SNPs in the pools versus those from previous individual genotyping. Estimates of nucleotide diversity and Tajima's D are higher when mapping to the Pool‐seq assembly versus when mapping to the *S. salar* reference. However, the relationships between values are largely consistent. We conclude that the Pool‐seq approach explored here is a cost‐effective way to gain basic population genomic insights in nonmodel species where a reference genome is lacking. The method is particularly suitable for exploring population divergence but might also be used to compare relative levels of genome‐wide diversity among populations.

## CONFLICT OF INTEREST

None declared.

## AUTHOR CONTRIBUTIONS

The biological research initiating this study was led by L.L., S.K., A.A., and N.R. They provided the questions and materials and designed the study guided by L.A., C.W., and C.J.R. S.K. analyzed the data with M.P.C.M., J.H., and V.E.K., supervised by C.W., V.E.K., and L.A. Graphs and statistical analyses were produced by S.K., assisted by A.A. S.K. also led the writing, with all authors contributing to and approving the submitted manuscript. The research was funded by grants to L.L.

## Data Availability

Data for this study are available in the Dryad repository: https://doi.org/10.5061/dryad.q1h4k0n. Population genomic scripts are available in Appendix S3.
