# Supplementary material for: Exploring a Pool‐seq‐only approach for gaining population genomic insights in nonmodel species
Source: Ecol Evol. 2019 Sep 26;9(19):11448–63. doi: 10.1002/ece3.5646 (PMC6802065; doi:10.1002/ece3.5646)
Supplement: Supplementary file 1 [file ECE3-9-11448-s001.pdf]

Exploring a Pool-seq only approach for gaining population genomic insights in non-model species

Sara Kurland, Christopher W. Wheat, Maria de la Paz Celorio Mancera, Verena E. Kutschera, Jason Hill, Anastasia Andersson, Carl-Johan Rubin, Leif Andersson, Nils Ryman, Linda Laikre

Appendix S1: Background on sympatric brown trout population pairs used in the present study

We study four populations (two population pairs) that inhabit small freshwater lakes in a remote, mountainous area within the Hotagen Nature Reserve, located at c. 700 m elevation in Jämtland County, central Sweden (Fig. 1). These lakes belong to a long-term monitoring project during which c. 100 individuals per lake have been collected annually since the mid-1980s and onwards (Jorde & Ryman, 1996; Laikre, Jorde, & Ryman, 1998; Palm & Ryman, 1999; Palm, Laikre, Jorde, & Ryman, 2003; Charlier, Palmé, Laikre, Andersson, & Ryman, 2011; Charlier, Laikre, & Ryman, 2012; Palmé, Laikre, & Ryman, 2013, Andersson et al. 2017a; b). Typically, 100 fish have been collected annually from each lake (including from Lakes Lilla Bävervattet, Stora Bävervattnet, Västra Trollsvattnet and Östra Trollsvattnet that were used for the present study; Fig 1). All samples have been stored in -80°C freezers since the time of collection.

Introduced sympatric populations

The introduced pair of populations was released into the Lakes Bävervattnen system, two interconnected lakes of < 1 km^2 at near 700 m elevation (Fig. 1). A waterfall separates these lakes from downstream waters inhabited by brown trout, and the lakes were inhabited only by Arctic charr (*Salvelinus alpinus)* prior to the introduction (Fig. S1). The released individuals originated from two separate populations showing diverse ecological features and that had been isolated from each other since the last glaciation (c. 5,000 - 9,000 years ago; Ryman et al., 1986; Palm & Ryman, 1999). One of the introduced populations (I) is from a hatchery population frequently used to stock waters in Jämtland County (Bergman, Halvarson, & Larsson, 1989). This hatchery strain originates from a wild population from Lake Kallsjön, a large lake of c. 155 km^2 at 380 m elevation that belongs to the River Indalsälven catchment, draining into the Baltic Sea. Fish in the Lake Kallsjön population were large, slow-growing and piscivorous, down-stream spawners with a presumed capacity for long distance migration (Ryman et al., 1986). Construction of hydro power plants in the1940s obstructed spawning and ultimately led to the wild populations' extinction in the 1980s. Population decline was noticed in the 1940s, and a hatchery population was created to compensate this loss. This hatchery population was initially maintained by contribution from wild spawners from River Kallströmmen, but as the wild population diminished this contribution dwindled, and since the 1950s the hatchery population is mainly upheld by hatchery reared fish. The other introduced population (II) originates from a landlocked population inhabiting Lakes Fälpfjälltjärnarna, two tiny lakes of < 1 km^2 at 645 m elevation. The fish here exhibit traits typical of small-mountain-lake populations; small body size, maturation at an earlier age and an insect based diet (Ryman et al., 1986).

Previous analyses have shown that the Lake Kallsjön and Lakes Fälpfjälltjärnarna populations exhibit significant allele frequency differences at multiple allozyme loci, particularly so at AGP-2, where the Lake Kallsjön population was close to fixation for one of the alleles at this locus (the so-called 100 allele) while in the Lakes Fälpfjälltjärnarna the 100 allele and another allele (called 50) occurred at equal frequencies (Palm & Ryman, 1999). Using the AGP-2 locus, spawners from each population were chosen to be homozygous for different alleles (100/100 vs. 50/50), thereby enabling discrimination of lineages after introduction (Palm & Ryman, 1999). According to hatchery records more than 100 potential breeders of both sexes of the Lake Kallsjön population were accessible for mating in the fall of 1978, and over 100,000 eggs were produced. For the Lakes Fälpfjälltjärnarna population, wild spawners were caught during the spawning season of 1978. The fish were transported to the hatchery, where they were genotyped and stripped. Of the 102 spawners genotyped, 6 females and 11 males were identified as homozygous for the 50 allele and used to produce offspring.

The introduction of the juveniles was conducted in late July in 1979 by releasing 1,000 fry from each population to two locations in the Lakes Bävervattnen system (Fig. S1; Ståhl & Ryman, 1982). The fish in these lakes have been monitored since 1988 primarily using allozymes but with subsets analyzed for mtDNA, microsatellites, and SNPs (Laikre et al. 1998; Palmé et al. 2013; Andersson et al. 2017a). After the release, 41 individuals could be classified based on their age and *AGP-2* genotype to either of the two released populations, all of which were used in the present study. The additional 59 fish used here were "pure" F1 (offspring from matings within populations as clarified from *AGP-2* genotype) randomly collected out of c. 700 available fish.

Naturally occurring sympatric populations

The Lakes Trollsvattnen system is comprised of a series of tiny interconnected mountain lakes and tributaries at the northernmost part of the River Indalsälven drainage system. The two main lakes (Östra and Västra Trollsvattnen) are oligotrophic, c. 0.1-0.17 km^2 in size and with a depth of mostly 1-2 m.

These lakes have been sampled since the 1980s and an initial analysis using 14 polymorphic allozyme loci revealed significant heterozygote deficiencies in both of the lakes (Jorde & Ryman, 1996). Further analyses confirmed population genetic structuring in the pooled material from both lakes (F_{IT}=0.042; Palmé et al., 2013), as well as within each of the two lakes separately (F_{IS}=0.038 and 0.044 for Östra and Västra Trollsvattnen, respectively). STRUCTURE analysis suggested two populations in the total material as well as within each lake regardless of assumptions of ancestry and allele-frequency models (Palmé et al., 2013). Considering individuals assigned to the clusters suggested by STRUCTURE at cut-offs for assignment between 0.5-0.9 yielded F_{ST} between 0.1- 0.2 (Palmé et al., 2013; Andersson et al., 2017a). However, in an analysis of 3,093 SNPs from a subset of the initially studied individuals, genetic differentiation is a fraction of that detected by allozymes (F_{ST} = 0.03; Andersson et al., 2017a). Based on analyses of phenotypic traits and diet, the populations in Lakes Trollsvattnen give no indication of trophic polymorphism or ecological niche separation (Andersson et al., 2017b). The fish used in the present study were a randomly chosen sample from c. 2,000 individuals with an

assignment probability of ≥ 0.8 to either of the two genetic clusters based on allozyme genotype (see Andersson et al., 2017a for more details).

Figure S1 Schematic illustration of sympatric populations I (hatchery source) and II (lake resident source), and their introduction to Lakes Bävervattnen.

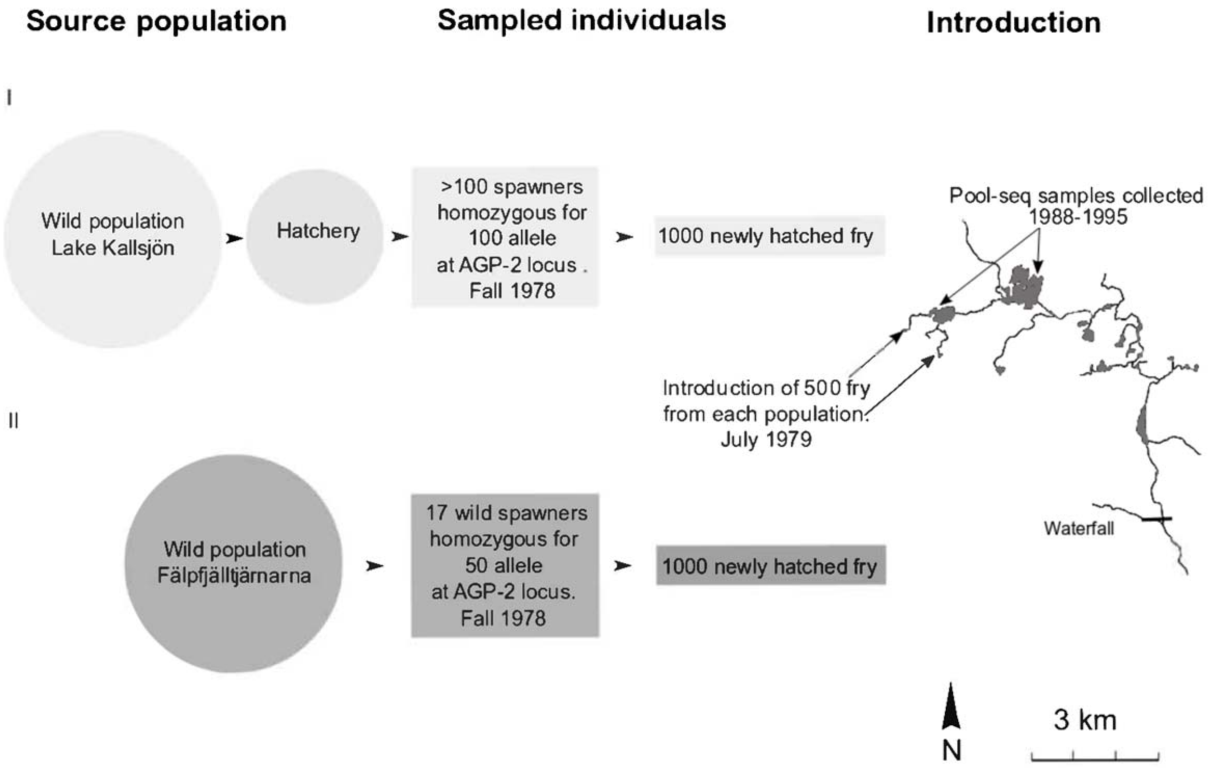

Literature cited

Andersson, A., Jansson, E., Wennerström, L., Chiriboga, F., Arnyasi, M., Kent, M. P., & Laikre, L. (2017a) Complex genetic diversity patterns of cryptic, sympatric brown trout (Salmo trutta) populations in tiny mountain lakes. Conservation Genetics, 18(5), 1213–1227. https://doi.org/10.1007/s10592-017-0972-4

Andersson, A., Johansson, F., Sundbom, M., Ryman, N., & Laikre, L. (2017b). Lack of trophic polymorphism despite substantial genetic differentiation in sympatric brown trout (*Salmo trutta*) populations. Ecology of Freshwater Fish, 26, 643-652. https://doi.org/10.1111/eff.12308

Bergman, W., Halvarson, J. & Larsson, M. 1989. Öringstammar i Sverige: Åtgärdsstudier för kompensation av fiskeskador, delrapport 2. (In Swedish). FUD Vattenfall, Vällingby.

Carruthers, M., Yurchenko, A. A., Augley, J. J., Adams, C. E., Herzyk, P., & Elmer, K. R. (2018). De novo transcriptome assembly, annotation and comparison of four ecological and evolutionary model salmonid fish species. BMC Genomics, 19:32, 1–17. https://doi.org/10.1186/s12864-017-4379-x

Charlier, J., Laikre, L., & Ryman, N. (2012). Genetic monitoring reveals temporal stability over 30 years in a small, lake-resident brown trout population. Heredity, 109(4), 246–253. https://doi.org/10.1038/hdy.2012.36

Charlier, J., Palmé, A., Laikre, L., Andersson, J., & Ryman, N. (2011). Census (NC) and genetically effective (Ne) population size in a lake-resident population of brown trout Salmo trutta. Journal of Fish Biology, 79(7), 2074–2082. https://doi.org/10.1111/j.1095-8649.2011.03124.x

Jorde, P., & Ryman, N. (1996). Demographic Genetics of Brown Trout (Salmo trutta) and Estimation of Effective Population Size from Temporal Change of Allele Frequencies. Genetics Society of America, (143), 1396–1381.

Laikre, L., Jorde, P. E., & Ryman, N. (1998). Temporal Change of Mitochondrial DNA Haplotype Frequencies and Female Effective Size in a Brown Trout (Salmo trutta) Population. Society for the Study of Evolution, 52(3), 910–915.

Palm, S., & Ryman, N. (1999). Genetic basis of phenotypic differences between transplanted stocks of brown trout. Ecology of Freshwater Fish, 8(3), 169–180.

Palm, S., Laikre, L., Jorde, P., & Ryman, N. (2003). Effective population size and temporal genetic change in stream resident brown trout (Salmo trutta, L.). Conservation Genetics, 4(3), 249–264. http://dx.doi.org/10.1023/A%3A1024064913094

Palmé, A., Laikre, L., & Ryman, N. (2013). Monitoring reveals two genetically distinct brown trout populations remaining in stable sympatry over 20 years in tiny mountain lakes. Conservation Genetics, 14(4), 795–808. https://doi.org/10.1007/s10592-013-0475-x

Ryman, N., Öhman, R., Ståhl, G., Nilsson, A, Lagercrantz, U. & Herlitz, A. 1986. Genetisk variation hos laxartad fisk - en snabbt försvinnande naturresurs: Information om genetisk forskning i Jämtland [Genetic variation in salmonid fishes - a rapidly declining natural resource: information on the scientific work in the lakes Bavervattnen area in Jämtland County, central Sweden] (in Swedish). Stockholm: Resta Grafiska AB.

Ståhl, G., & Ryman, N. (1982). Simple Mendelian inheritance at a locus coding for a – glycerophosphate dehydrogenase in brown trout (Salmo trutta). Hereditas, 96, 313-315. https://doi.org/10.1111/j.1601-5223.1982.tb00865.x
