# Supplementary material for: Exploring a Pool‐seq‐only approach for gaining population genomic insights in nonmodel species
Source: Ecol Evol. 2019 Sep 26;9(19):11448–63. doi: 10.1002/ece3.5646 (PMC6802065; doi:10.1002/ece3.5646)
Supplement: Supplementary file 2 [file ECE3-9-11448-s002.pdf]

Exploring a Pool-seq only approach for gaining population genomic insights in non-model species

Sara Kurland, Christopher W. Wheat, Maria de la Paz Celorio Mancera, Verena E. Kutschera, Jason Hill, Anastasia Andersson, Carl-Johan Rubin, Leif Andersson, Nils Ryman, Linda Laikre

Appendix S2: Coverage calculations, RNA-seq accessions and diversity estimates

Coverage calculations of Pool-seq data

Read depth histograms were calculated and assessed for Pool-seq data mapped to the two reference assemblies to set appropriate limits for population genetic calculations, in order to improve accuracy of estimates by excluding low and high coverage regions. This was done in BEDTools genomecov version 2.25.0 (Quinlan & Hall, 2010) for reads mapped to the *S. trutta* draft assembly and the *S. salar* reference genome.

Estimates of diversity within population pools are comparably more sensitive to sequencing errors than estimates of population divergence (Kofler et al., 2011a; 2016). Calculations were made in POPOOLATION version 1.2.2 (Kofler et al., 2011a) per population pool from one mpileup file per population pool, enabling pool-specific coverage limits assessed from the read depth histograms. To standardize for sequencing errors, mpileup files were subsampled to uniform coverage without replacement using the subsample-pileup.pl script (Kofler et al., 2011a) with a target coverage equal to the pool-specific mode of the read depth distribution and omitting sites with coverage that exceeded the mode plus half of the mode. The same coverage limits were chosen for estimates in POPOOLATION (47X ± 24X, 46X ± 23X, 50X ± 25X, and 57X ± 29X for the introduced population pools I and II, and the natural population pools A and B, respectively, mapped to the *S. trutta* assembly).

POPOOLATION2 (Kofler, Pandey, & Schlötterer, 2011b) enables pairwise comparisons (e.g., F_{ST}) where maximum coverage can be tailored to individual population pools, but where the same minimum coverage threshold is set for the included population pools. Pairwise comparisons were conducted separately for the introduced and natural pair. First, the mode of the read depth histogram was determined for each population pool. A lower limit was estimated for each pair by taking the average of half of the mode for the two population pools (23X and 27X for the introduced and natural pair, respectively, mapped to the *S. trutta* assembly). The population pool-specific upper limit was defined by adding the difference between the pool-specific mode and the averaged lower limit to the mode. The resulting confidence limits surrounding the modes for the introduced population pools I and II, and natural population pools A and B mapped to the *S. trutta* assembly were 47X ± 23X, 46X ± 23X, 50X ± 27X, and 57X ± 27X, respectively (Fig. S2).

The upper and lower coverage limits for the pool-seq data mapped to the *S. salar* reference were set the same way as described above for POPOOLATION (41X ± 21X, 40X ± 20X, 44X ± 22X, and 51X ± 26X for the introduced populations I and II, and natural populations A and B, respectively) and POPOOLATION2 (41X ± 20, 40X ± 20, 44X ± 24, and 51X ± 24 for the introduced populations I and II, and natural populations A and B, respectively; Fig. S3).

Testing window sizes for F_{ST} calculations

The fixation index (F_{ST}; Nei, 1973) was estimated for each population pair in POPOOLATION2 version 1201 (Kofler et al., 2011b) and calculated for non-overlapping windows to avoid increased stochastic error rates associated with small window size (Kofler et al. 2011b). A range of window sizes was tested (1 bp, 100 bp, 500 bp, 1 kb, and 5 kb) as well as various minimum proportions of a window being within coverage limits (50%, 80%, 90%, and 100%). 1 kb and 5 kb windows yielded very few observations and therefore proved to be too large for the *S. trutta* assembly. F_{ST} was similar across 100-500 bp windows when at least 80-90% of the window was covered (Table S3). Thus, 500 bp windows and only including windows with > 90% coverage was deemed appropriate to minimize stochastic errors without excluding too much data. For simplicity of comparisons, the same settings were chosen for reads mapped to *S. salar* as *S. trutta* and when calculating F_{ST} across coding and non-coding regions.

Tables S1-S3

Table S1 Brown trout RNA-seq data (NCBI bio project PRJNA419712) used to assemble the *S. trutta* proteome (published in Carruthers et al., 2018).

SRA study	Library_Name	Run
**SRS2713411**	St-500-Bw3_S7	SRR6321796
**SRS2713410**	St-500-Bw4_S8	SRR6321795
**SRS2713409**	St-500-Bw2_S6	SRR6321797
**SRS2713408**	St-500-Bw1_S5	SRR6321798
**SRS2713403**	St-500-Dp2_S2	SRR6321803
**SRS2713402**	St-500-Dp1_S1	SRR6321804
**SRS2713391**	St-500-Dp3_S3	SRR6321815
**SRS2713390**	St-500-Dp4_S4	SRR6321816

Table S2 BAM file statistics from QualiMap for Pool-seq data from population BVA mapped to the MESPA genome, using bbmap, bwa mem and NextGenMap using default settings. Analysis limited to properly paired-end reads and reads of mapping quality > 20.

Mapping statistics	bbmap	bwa mem	NextGenMap
Number of mapped paired reads	236,810,028	256,750,954	140,927,744
Mean coverage (Standard Deviation)	89 (1,491)	72 (1,404)	44 (452)
Mean mapping quality[1]	38	56	57
General error rate[2]	13.2%	2.7%	1.4%

[1] Mapping algorithms calculate mapping quality different from each other.
[2] Computed as a ratio of total collected edit distance to the number of mapped bases

Table S3 Average pairwise F_{ST} between introduced and natural populations, respectively, estimated from POPOOLATION2 using different window sizes and different fractions of windows within coverage limits, from Pool-seq data mapped to the *S. trutta* assembly and *S. salar* genome, respectively. The number of (*n*) windows is specified.

Reference	Window size (bp)	Fraction covered	Pairwise comparison	*n* (windows)	F_{ST}
S. trutta	100	0.8	Introduced	1,062,248	0.10
		0.8	Natural	1,062,664	0.02
		0.9	Introduced	748,582	0.11
		0.9	Natural	740,368	0.03
		1	Introduced	639,392	0.12
		1	Natural	633,219	0.03
	500	0.8	Introduced	380,326	0.11
		0.8	Natural	377,474	0.03
		0.9	Introduced	278,077	0.13
		0.9	Natural	274,930	0.03
		1	Introduced	83,436	0.15
		1	Natural	81,962	0.03
S. salar	100	0.8	Introduced	2,826,318	0.13
		0.8	Natural	2,868,512	0.03
		0.9	Introduced	1,982,869	0.14
		0.9	Natural	2,008,148	0.03
		1	Introduced	1,635,420	0.14
		1	Natural	1,691,080	0.03
	500	0.8	Introduced	1,084,190	0.15
		0.8	Natural	1,137,375	0.03
		0.9	Introduced	661,711	0.15
		0.9	Natural	732,377	0.03
		1	Introduced	91,510	0.16
		1	Natural	111,001	0.03

Figures S2-S6

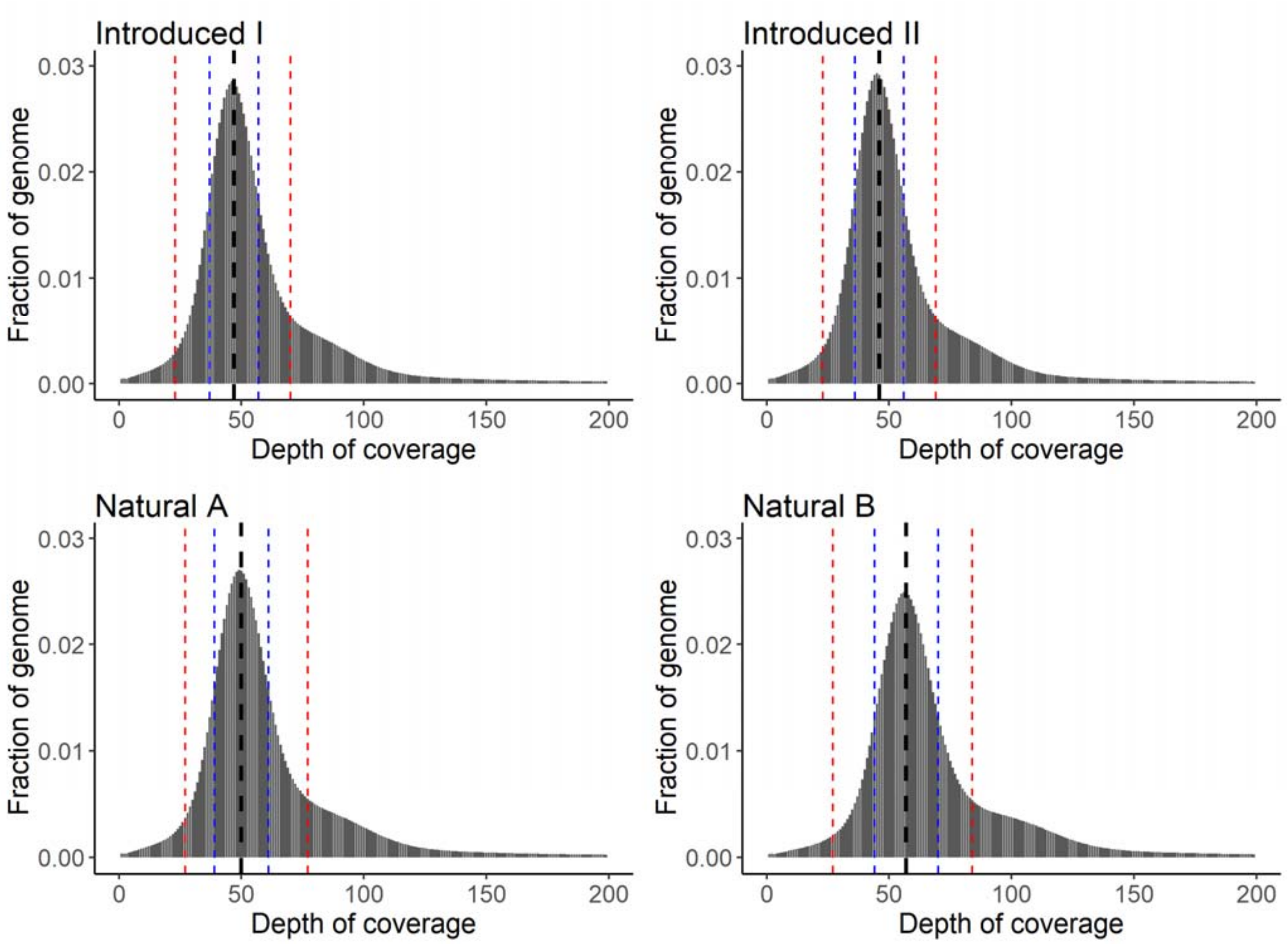

Figure S2 Histograms of depth of coverage in each population pool mapped to the S. trutta genome assembly with mode (black dotted lines) and depth thresholds used in POPOOLATION (blue dotted lines) and POPOOLATION2 (red dotted lines), respectively.

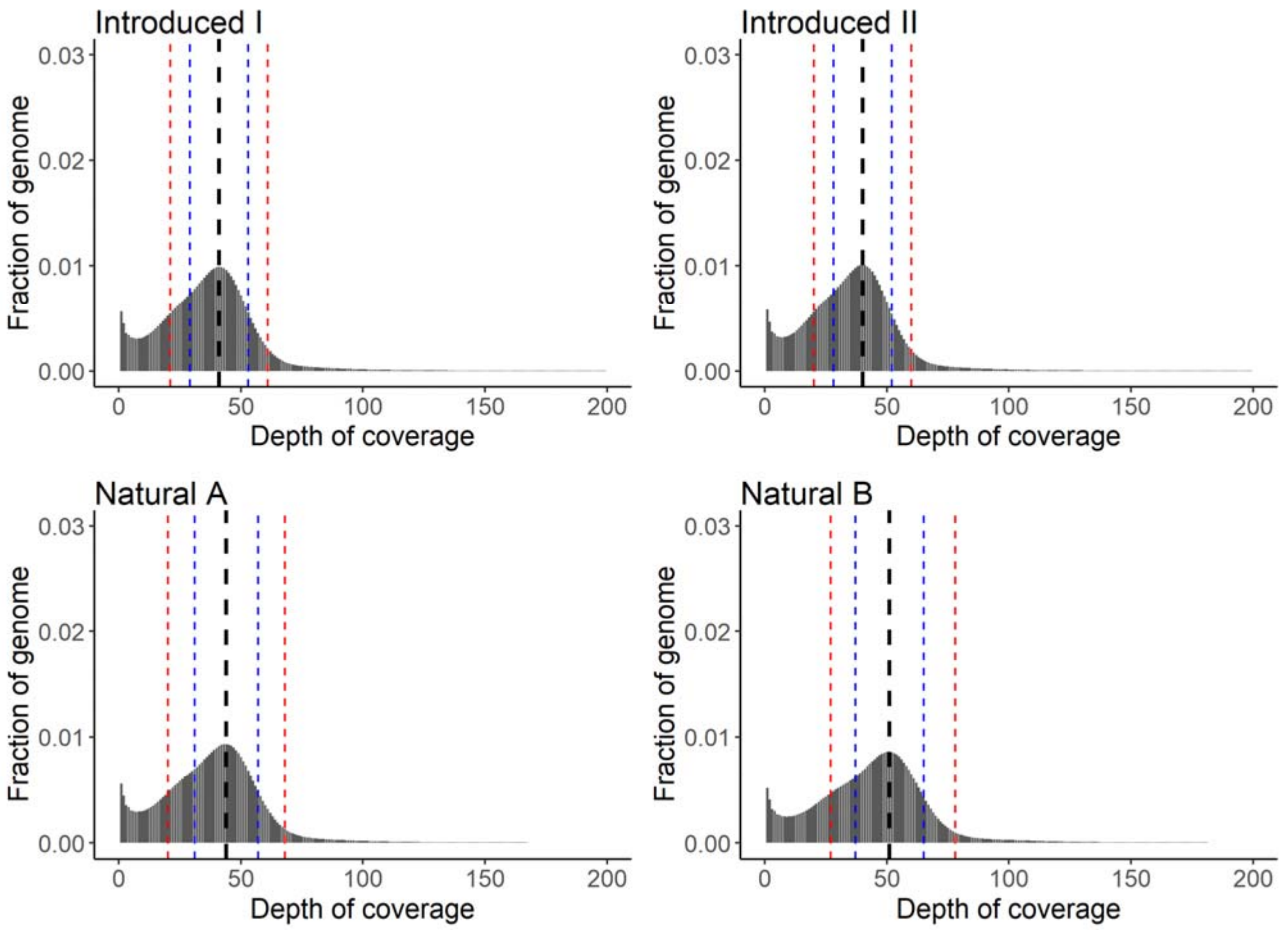

Figure S3 Histograms of depth of coverage in each population pool mapped to the *S. salar* reference genome with mode (black dotted lines) and depth thresholds used in POPOOLATION (blue dotted lines) and POPOOLATION2 (red dotted lines), respectively.

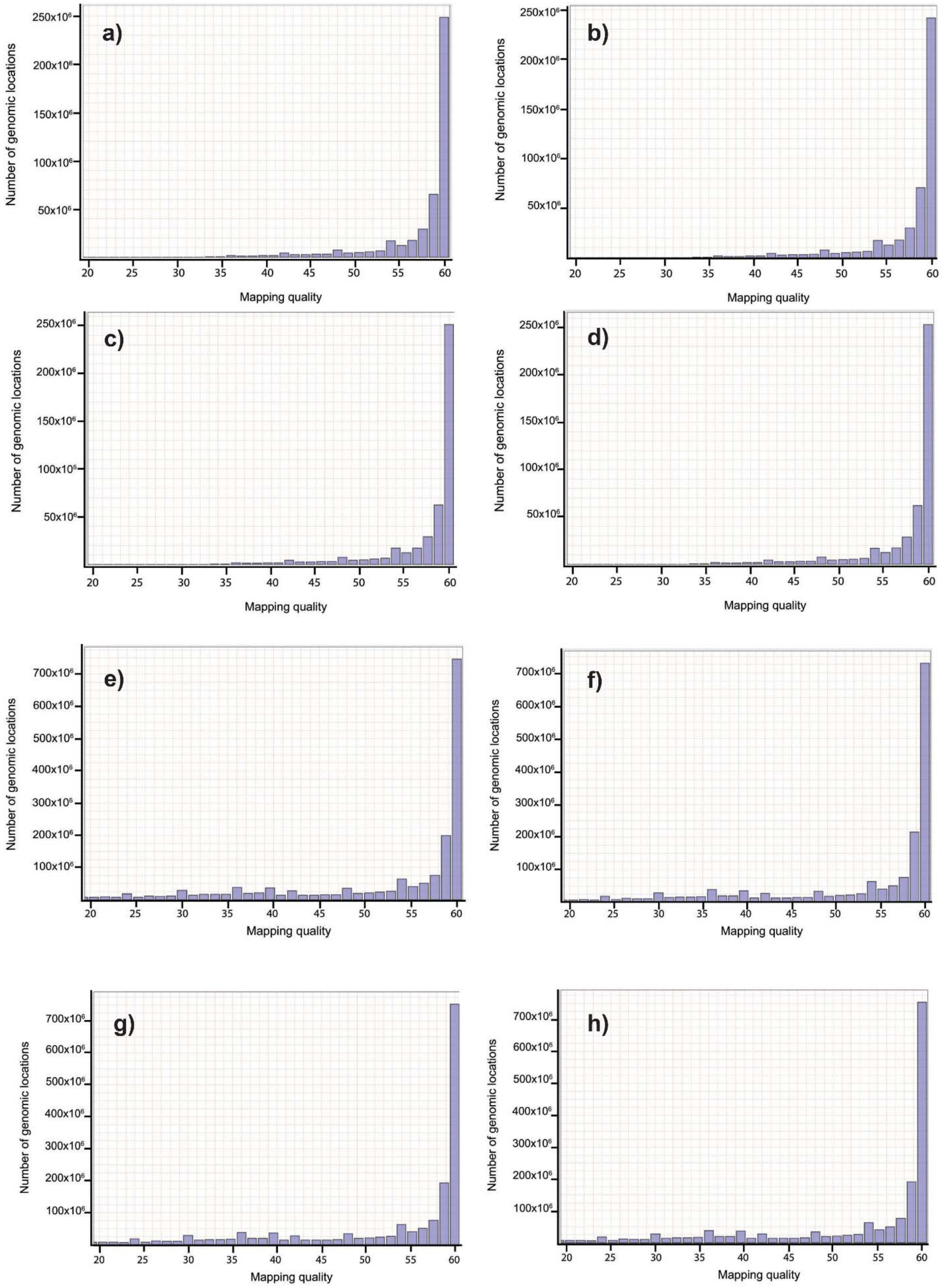

Figure S4 Mapping quality from bwa mem, filtered for minimum base quality 20 and mapping quality 20. (a) Naturally sympatric population A mapped to the Pool-seq based, MESPA generated *Salmo trutta* assembly. b) Introduced population I mapped to the *S. trutta* assembly, c) Naturally sympatric population B mapped to the *S. trutta* assembly, d) Introduced population II mapped to the *S. trutta* assembly. e)-h) The same populations mapped to the Atlantic salmon (*Salmo salar*) reference genome.

a)

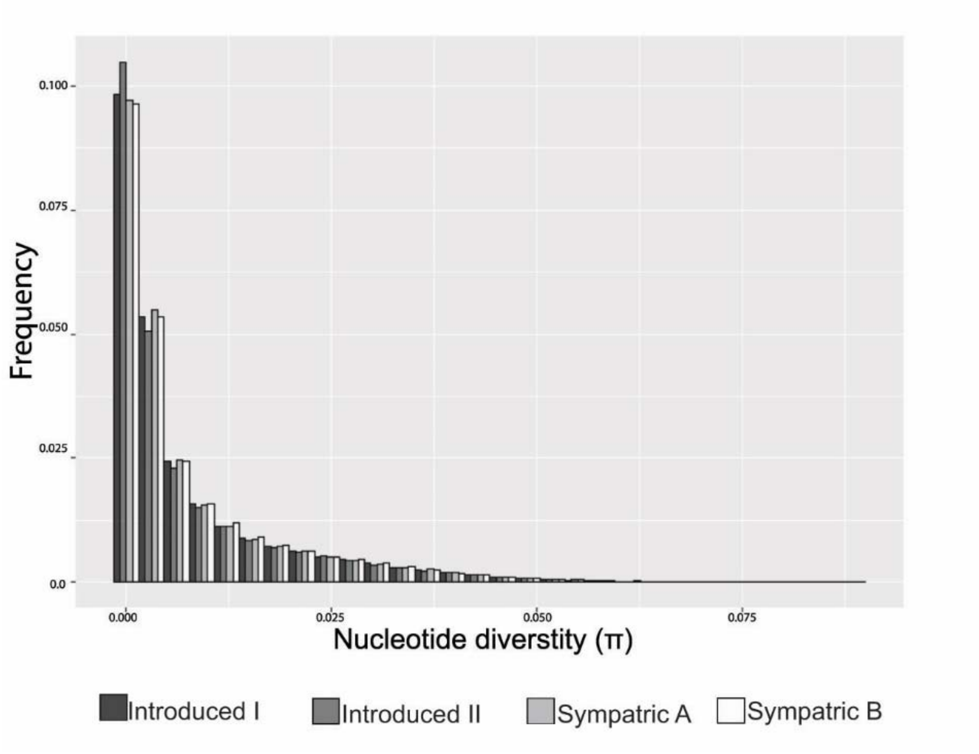

b)

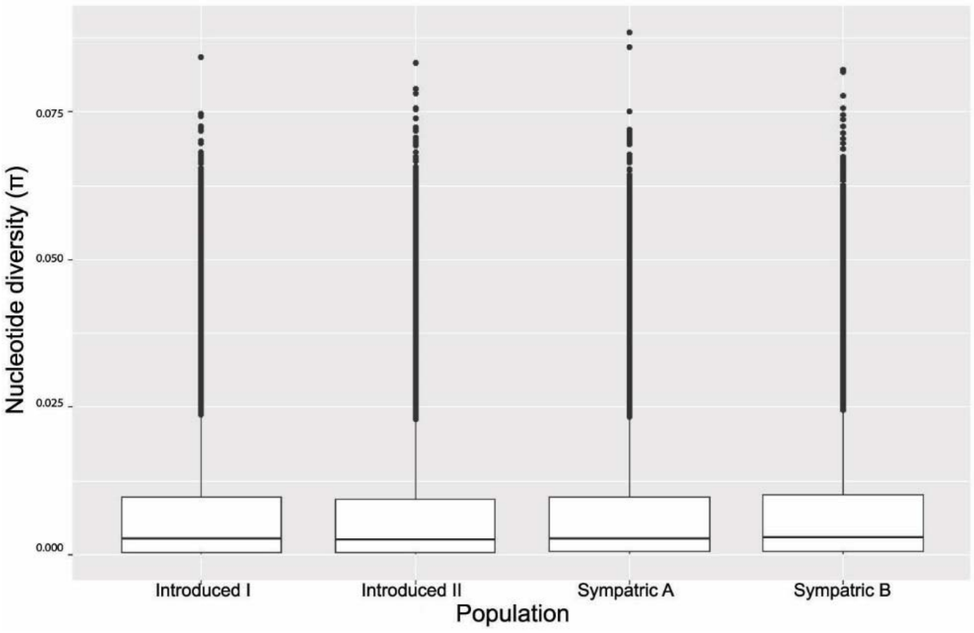

Figure S5 Frequency distribution (a) and boxplot (b) (with outliers as black dots) of nucleotide diversity (π) within coding regions per 500 bp windows within each population pool mapped to the *S. trutta* assembly.

a)

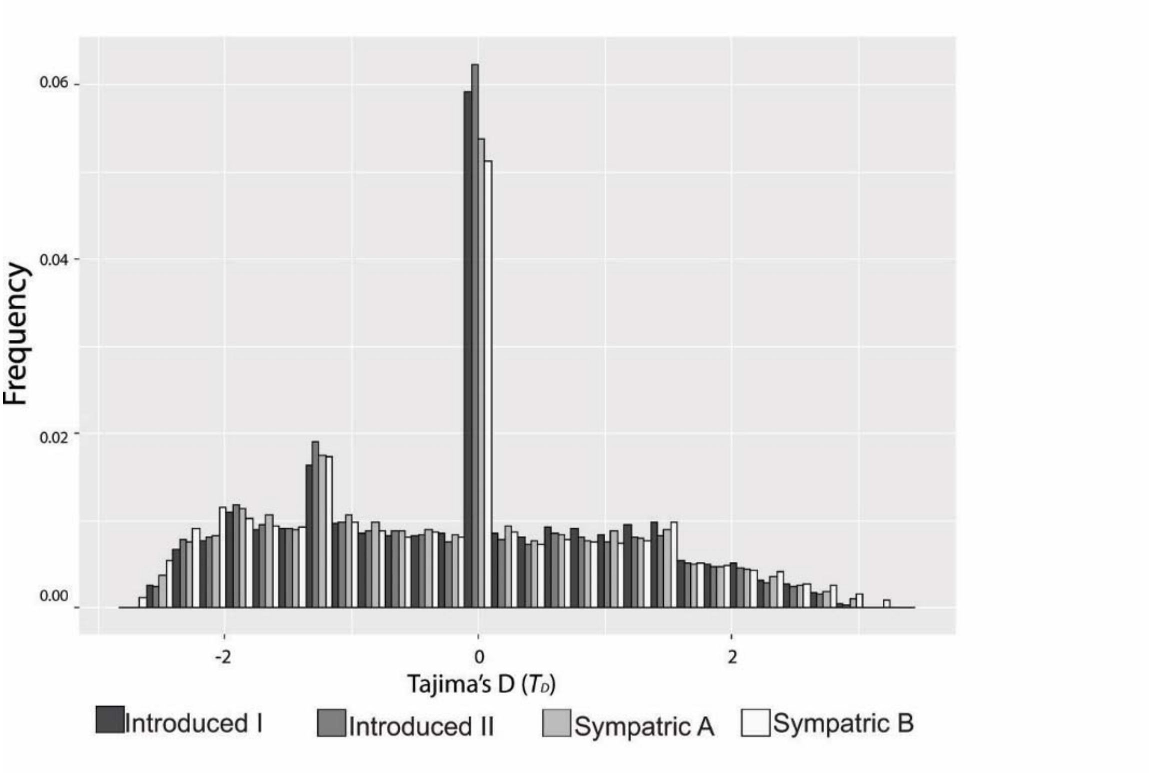

b)

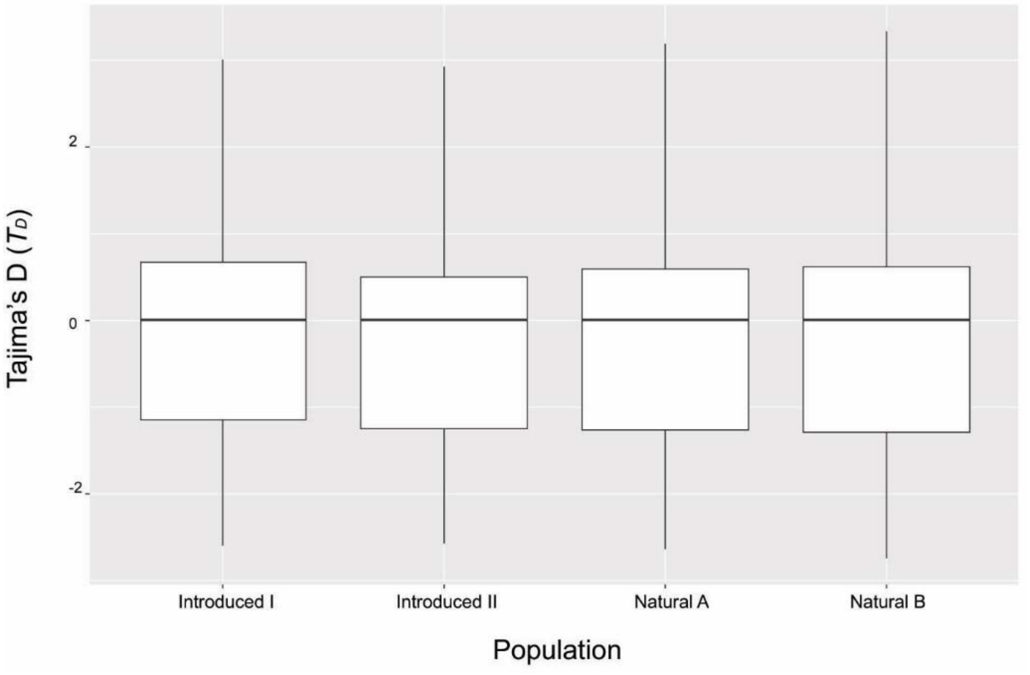

Figure S6 Frequency distribution (a) and boxplot (b) (with outliers indicated by black, vertical lines) of Tajima's D (T_D) within coding regions per 500 bp windows within each population pool mapped to the *S. trutta* assembly.

References

García-Alcalde, F., Okonechnikov, K., Carbonell, J., Cruz, L. M., Götz, S., Tarazona, S., Dopazo, J., Meyer, T. F., & Conesa, A. (2012). Qualimap: evaluating next-generation sequencing alignment data. Bioinformatics, 28(20), 2678-2679.

Kofler, R., Orozco-ter Wengel, P., de Maio, N., Pandey, R. V., Nolte, V., Futschik, A., Schlötterer, C. (2011a). PoPoolation: A toolbox for population genetic analysis of next generation sequencing data from pooled individuals. PLoS ONE, 6. https://doi.org/10.1371/journal.pone.0015925

Kofler, R., Pandey, R. V., & Schlötterer, C. (2011b). PoPoolation2: identifying differentiation between populations using sequencing of pooled DNA samples (Pool-Seq). Bioinformatics, 27(24), 3435-3436.

Kofler, R., Langmüller, A. M., Nouhaud, P., Otte, A. K., & Schlötterer, C. (2016). Suitability of different mapping algorithms for genome-wide polymorphism scans with Pool-Seq data. G3: Genes, Genomes, Genetics, 6, 3507-3515. https://doi.org/10.1534/g3.116.034488

Quinlan, A. R., & Hall, I. M. (2010). BEDTools: A flexible suite of utilities for comparing genomic features. Bioinformatics, 26, 841–842. https://doi.org/10.1093/bioinformatics/btq033
