# Supplementary material for: Exploring a Pool‐seq‐only approach for gaining population genomic insights in nonmodel species
Source: Ecol Evol. 2019 Sep 26;9(19):11448–63. doi: 10.1002/ece3.5646 (PMC6802065; doi:10.1002/ece3.5646)
Supplement: Supplementary file 3 [file ECE3-9-11448-s003.pdf]

Exploring a Pool-seq only approach for gaining population genomic insights in non-model species

Sara Kurland, Christopher W. Wheat, Maria de la Paz Celorio Mancera, Verena E. Kutschera, Jason Hill, Anastasia Andersson, Carl-Johan Rubin, Leif Andersson, Nils Ryman, Linda Laikre

Appendix S3: Scripts used for population genetic/genomic analyses

1) Between-population statistics
Software: popoolation v2/1201
1.1) Fst
```
perl /popoolation2/1201/fst-sliding.pl --input ${1}.sync --output ${1}.fst.txt
--suppress-noninformative --min-covered-fraction 0.0 --min-count ${mincount}
--window-size 500 --step-size 500 --min-coverage ${mincov}
--max-coverage ${maxcov} --pool-size 100
```

1.2) Allele frequency differences
```
perl /popoolation2/1201/snp-frequency-diff.pl --input ${1}.sync --output-prefix ${1}
--min-count 3 --min-coverage ${mincov} --max-coverage ${maxcov}
```

2) Within-population statistics
Software: popoolation v1.2.2

2.1) Split the mpileup file into one file per population
```
cd ${mpileupdir}
# first population
cut -f 1-6 ${popA}_${popB}.${refname}.q20_Q20_maskedindel.mpileup >
${popA}.${refname}.q20_Q20_maskedindel_split.mpileup
# second population
cut -f 1-3,7-9 ${popA}_${popB}.${refname}.q20_Q20_maskedindel.mpileup >
${popB}.${refname}.q20_Q20_maskedindel_split.mpileup
```

2.2) Subsample mpileup files to even depth
```
perl /popoolation/1.2.2/basic-pipeline/subsample-pileup.pl --input ${1}.mpileup
--output ${1}_subsampled.mpileup --min-qual 20 --fastq-type sanger
--method ${method} --target-coverage ${targetcov} --max-coverage ${maxcov}
```

2.3) Tajima's D and pi
```
perl /popoolation/1.2.2/Variance-sliding.pl --input ${1}.mpileup --output ${1}.TajD.txt
--measure D --fastq-type sanger --min-qual 20 --min-covered-fraction 0.0
--min-count 2 --window-size 500 --step-size 500 --min-coverage ${mincov}
--max-coverage ${maxcov} --pool-size 100

perl /popoolation/1.2.2/Variance-sliding.pl --input ${1}.mpileup --output ${1}.pi.txt
--measure pi --fastq-type sanger --min-qual 20 --min-covered-fraction 0.0
--min-count 2 --window-size 500 --step-size 500 --min-coverage ${mincov}
--max-coverage ${maxcov} --pool-size 100
```

3) Filtering of population statistics output

The output files obtained from point 2.3 above provide columns including window start position, number of SNPs per window, percentage of coverage with data per window, and the measure obtained for this window. Calculations to obtain the total numbers of SNPs and windows, mean values of Tajima's D, pi and Fst as well as 95% confidence intervals were performed in R (R Core Team, 2017).
